# Potential unsatisfiability of cyclic constraints on stochastic biological networks biases selection towards hierarchical architectures

**DOI:** 10.1098/rsif.2015.0179

**Published:** 2015-07-06

**Authors:** Cameron Smith, Ximo Pechuan, Raymond S. Puzio, Daniel Biro, Aviv Bergman

**Affiliations:** 1 Department of Systems and Computational Biology, Albert Einstein College of Medicine, 1301 Morris Park Avenue, Bronx, NY 10461, USA; 2 Dominick P. Purpura Department of Neuroscience, Albert Einstein College of Medicine, 1301 Morris Park Avenue, Bronx, NY 10461, USA; 3 Department of Pathology, Albert Einstein College of Medicine, 1301 Morris Park Avenue, Bronx, NY 10461, USA; 4 Santa Fe Institute, 1399 Hyde Park Road, Santa Fe, NM 87501, USA

**Keywords:** biological networks, evolution, genotype–phenotype map, systems biology

## Abstract

Constraints placed upon the phenotypes of organisms result from their interactions with the environment. Over evolutionary time scales, these constraints feed back onto smaller molecular subnetworks comprising the organism. The evolution of biological networks is studied by considering a network of a few nodes embedded in a larger context. Taking into account this fact that any network under study is actually embedded in a larger context, we define network architecture, not on the basis of physical interactions alone, but rather as a specification of the manner in which constraints are placed upon the states of its nodes. We show that such network architectures possessing cycles in their topology, in contrast to those that do not, may be subjected to unsatisfiable constraints. This may be a significant factor leading to selection biased against those network architectures where such inconsistent constraints are more likely to arise. We proceed to quantify the likelihood of inconsistency arising as a function of network architecture finding that, in the absence of sampling bias over the space of possible constraints and for a given network size, networks with a larger number of cycles are more likely to have unsatisfiable constraints placed upon them. Our results identify a constraint that, at least in isolation, would contribute to a bias in the evolutionary process towards more hierarchical -modular versus completely connected network architectures. Together, these results highlight the context dependence of the functionality of biological networks.

## 1. Introduction

Probabilistic models of biological networks serve as a bridge between theory and experiment. On the one hand, parameters in a probabilistic model can be fit to data obtained by measuring the levels of each variable. For example, in gene-regulatory networks, gene expression can be measured using microarray or sequence census methods [1–3]. On the other hand, one can model a biological network as a deterministic or stochastic reaction network which tracks levels of each molecule [4,5]. From the solution to this latter kind of model, one can then obtain theoretical predictions for the parameters of the probabilistic model in terms of reaction rates. Comparison of the parameters fitted from data with the predicted values serves as a means for comparing theory with experiment and can serve as a starting point for improving the theory or for designing future experiments [6].

An important feature of experimental science is that it involves partial information. In the course of a single measurement, one typically is not able to observe a biological network in its entirety. Rather, one observes a subnetwork at a time and only obtains a more complete picture by later combining these partial views. This contrasts with theory, where one makes a representation of a closed system that provides explicit values for all quantities of interest. In order for a probabilistic model to serve its purpose, it should also accomodate partial information and thus we will explicitly consider the effects of (i) carving out a subnetwork from its context and (ii) coarse-graining observables. Observables representing partial information will generally arise in situations where a system is interacting with another system. This situation arises in the context of interpreting the potential existence of modular substructure within biological network data deriving from any given organism as well as with respect to the interactions between an organism and its environment.

Inconsistency arises when a network context places more constraints on a subnetwork than it is capable of satisfying. The impact of this issue on genetic interactions has been considered previously in the context of population genetics [7]. We exhibit a method of checking for such consistency and evaluating its likelihood of arising in the context of building probabilistic models of biological networks. When apparent inconsistency is observed, it must arise from the network context interacting with only partial information of the states of a given subnetwork. This would indicate that information about the network context must be included in order to maintain a consistent model of the system.

In §2, we describe the relationship between representations of biological networks and an abstraction of these referred to as network architecture that indicates the manner in which a subset of a network is connected to its context. We explain the connection between stochastic process models of biological networks and a generalization of the genotype–phenotype map applying to arbitrary biological networks referred to as network–network state maps in §3. Sections 4–6 contain examples of the underlying mathematical justification for our claims (more details of which are provided in the electronic supplementary material), and they can be skipped by readers who are primarily interested in the intuitive implications of our analysis. In §4, we introduce the concept of network modules and define probability distributions over their states. Sections 5 and 6 describe the different compatibility conditions that arise for different biological network architectures and demonstrate how these compatibility conditions lead to a set of inequalities determining a space of probability distributions for each network architecture. Sections 7 and 8 examine these constraints for the example of the three-cycle network architecture. Section 9 computes the likelihood of unsatisfiable constraints for all biological network architectures on four variables that possess cycles. Finally, §10 explains implications for the evolution of biological network architectures of the result that networks with a larger number of cycles are more likely to have unsatisfiable constraints placed upon them.

## 2. Environments of biological networks as abstract contexts

Most studies of biological networks focus on one type of variable in isolation. For example, many studies focus on one of metabolic networks, protein–protein interaction networks, signalling networks, gene-regulatory networks, or population and community dynamics in the context of ecological networks. A true biological network involves all of these acting together to produce biological phenomena at all scales. Models that integrate information about biological networks, rather than focusing exclusively on particular types of molecules, will likely become more common in the near future [8–10]. The systems biology graphical notation (SBGN) supports the ability to express many of these networks within the context of a single formalism ([11], figure 1). Even when the different types of biological variables are combined into a single network, it is impossible to study all variables simultaneously. As a result, it is always the case that a subnetwork is selected for investigation and the remainder of the network is treated as an *environment* or *context*. In figure 1, we show the SBGN process form of six simple examples of biological networks. In each case, we have selected a subset of variables that form a subnetwork as an example of how one might proceed in the investigation of a particular biological system. Once such a subnetwork is chosen, it is possible to abstract away the variables that are not part of the subnetwork. This is represented by the abstract influence (AI) network for each simple example on figure 1*b*. The transformation from SBGN to the AI network is given simply by collapsing the disconnected components of the ancestors of each node in the focal subnetwork into single AI nodes. This results in a bipartite graph that captures the dependencies among the environmental factors as experienced by the subnetwork and nothing more.

**Figure 1.**
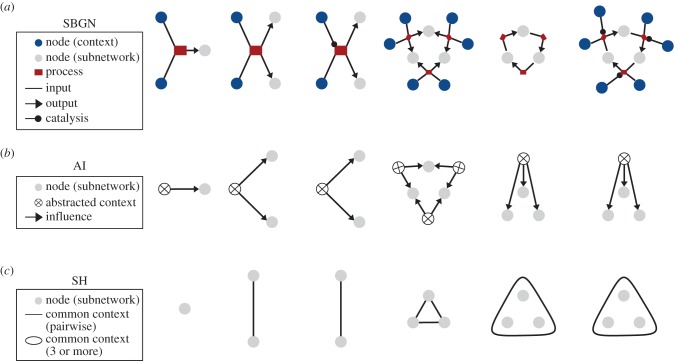
Abstract influence (AI) representation of biological networks. (*a*) The SBGN is capable of representing arbitrary biological networks including processes that involve metabolites, signalling molecules, genes and enzymes [11]. Only a fragment of the SBGN language, where all nodes have equivalent types, is indicated here. (*b*) We abstract from the SBGN representation of a biological network to a graph representing the AI graph indicating coupling among a subset of the entities present in a biological network. (*c*) For economy of representation, we use a short hand (SH) hypergraph to denote the AI graph. The topology of the AI and SH graphs are equivalent and this is what we refer to as network architecture.

This AI graph is precisely equivalent to an undirected hypergraph if one considers each of the AI nodes as a hyperedge containing all of the nodes to which it connects. This is shown as the SH graph in figure 1*c* for each of the simple examples of the SBGN form of biological networks. Considering all possible hypergraphs of this kind is equivalent to examining all possible environmental dependency structures the subnetwork could be subjected to. Because the AI is fundamental to understanding how subnetworks depend upon their contexts, it is the structure of the AI and equivalent SH graphs that we refer to as *network architecture* throughout the paper. We note from this perspective that cycles in the SBGN representation of the biological network do not result in corresponding cycles in the AI graph and vice versa. For instance, in example four of figure 1, there are no cycles in the SBGN representation of the biological network, whereas a single cycle exists in the hypergraph representation of the AI graph. Furthermore, in example six, there is a cycle in the SBGN representation, whereas there is no cycle in the hypergraph representation of the AI.

More precisely, the collection of variables comprising the subnetwork under consideration is referred to as *L*. The different subsets, *O*, of biological variables, *L*, making up the hypergraph representation of the AI are each referred to as modules. A biological network architecture, 𝒢, may then be represented by a subset of all possible such modules subject to two conditions (see electronic supplementary material, §S2). The first represents the fact each variable of the focal subnetwork must be included in at least one module. The second represents the fact that any pair of constraints that are imposed upon overlapping sets of variables must agree on those overlapping variables. In expressing the latter condition, all of the information present in a collection of lower order constraints can be expressed as an effective higher order constraint if any such higher order constraint exists at all. So, if there is a constraint that is imposed simultaneously upon two distinct variables and another independent constraint imposed upon only the first of the two variables, this situation can be expressed in terms of a single constraint on both of the two variables.

When there is a relatively larger degree of independence in the network context compared with the subnetwork, it is possible for inconsistency to arise. One canonical example of such inconsistency arises in the study of ferromagnetism via the Ising model on a triangular lattice where so-called *frustration* arises in the couplings among the magnetic dipole moments of three nearest neighbour atomic spins [12–14]. In this example, the underlying lattice or graph represents interactions among the spins of atomic nuclei according to their spatial proximity. As we have described, in our model, the network architectures to which we refer represent the manner in which the network context places constraints upon a subnetwork. Inconsistency is likewise capable of arising if there is a cycle in the hypergraph representing this network architecture.

## 3. Coarse-graining dynamic network states as a generalization of genotype–phenotype maps

**Figure 2.**
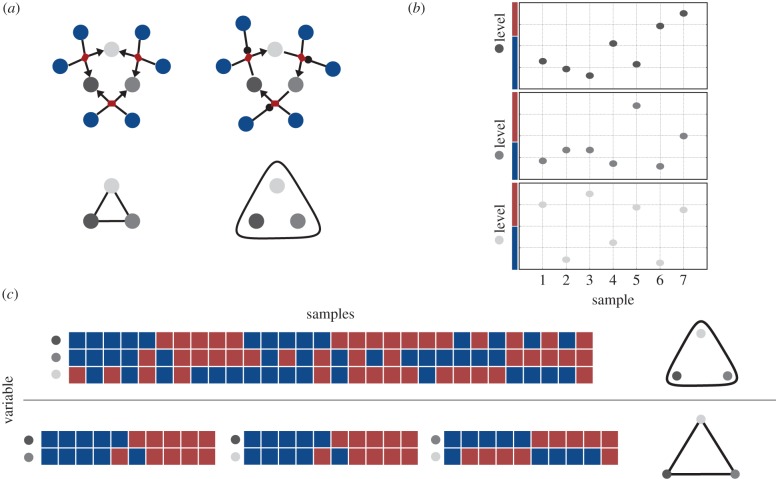
Coarse-graining of biological network data. (*a*) SBGN (top) and SH (bottom) representation of two different biological networks. (*b*) Example binary coarse-graining of biological network data. For each sample, a measurement is taken for all three variables in the focal subnetwork. The levels are binned into one of two classes represented by the red and blue bars representing relatively high and low levels, respectively. (*c*) Heat map representation of coarse-grained data under the assumption of two different network architectures. The samples on top and the associated measurement structure correspond to the case where constraints are placed on all three variables by a single element of the network context (figure 6, top row). The bottom represents the case where all three pairs are each independently constrained by elements of the network context (figure 6, bottom row).

Figure 2*a* shows a simplified representation of two different biological networks, the correlation strengths among whose variables are not known but are to be derived from observation of the levels of the entities corresponding to each variable. For example, in the context of a gene-regulatory network, the amount of a given transcript present in a cell can be binned into a smaller number of discrete classes by setting a collection of thresholds on the original dataset. If only a single threshold is given, then the data can be binned into two classes depending upon whether or not the original measurement surpasses the given threshold in figure 2*b*. The time series that results from such observations can be used to infer various statistics that characterize the dynamics of a biological network such as correlations between pairs of variables.

If a large enough number of thresholds is available to distinguish among all possible counts of the variables under investigation, then this observational protocol becomes complementary to mechanistic models. There may be several sources for stochasticity in the dynamics including small numbers of the causal molecules and products as well as environmental fluctuations upon which these dynamics are conditioned [15–23]. Regardless of the fundamental nature of biological networks with respect to their potential stochasticity, empirical observations are usually regarded in a statistical manner, and thus we focus here on stochastic models. Mathematically, such a model may take the form of a Markov chain whose dynamics are governed by a master equation for probability distributions over molecule counts. For example, in the case of a three variable network, the master equation takes the form
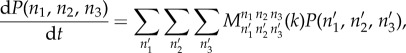
where *P*(*n*_1_, *n*_2_, *n*_3_) gives the probability of observing *n*_1_, *n*_2_ and *n*_3_ molecules of each of the three variables, respectively, and *M*(*k*) is a Markov transition rate matrix that depends upon some rate functions *k* that are determined by the network architecture and the dynamics of the interactions. The solution to this equation will converge towards a stationary distribution *P*_s_ in the limit of long times. Any environmental variable having a characteristic time scale longer than that of the variables in the focal subnetwork would not be sensitive to transients and would only exhibit control over or be influenced by this stationary distribution.

Interactions between variables may be mediated by a coarse-graining over counts of each variable using a function that maps the states representing molecule counts as vectors of natural numbers into some other variables. For example, if *n_i_* are natural numbers, then a function *f* taking any number less than or equal to some threshold *T* to 0 and any number greater than *T* to 1 is a very simple example of such a coarse-graining. For this specific form of the coarse-graining function *f*, the coarse-grained stationary probability distribution takes the form
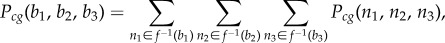
where *b*_1_, *b*_2_, *b*_3_ ∈ {0,1}. It is also possible to consider the case where each variable is coarse-grained according to a different threshold and into a different number of classes. An abstract algebraic formulation of the coarse-graining process is provided in electronic supplementary material, §S4.

The most familiar example of such a coarse-graining process in biology is the genotype–phenotype map. The genotype of an organism has a relatively straightforward definition in terms of the sequence of nucleotides comprising its genome. Phenotypes, on the other hand, can be described at different levels of organization [24,25]. The concept of phenotype was initially defined at the level of macroscopically observable physical characteristics such as shape, size, colour and various combinations thereof [26]. However, since the advent of molecular biology, an example of a lower level mapping upon which the higher level map from molecular states to macroscopic phenotypes depends is the dynamic phenomenon that can be described by measuring the transcription states of all genes comprising an organism's genome. These expression levels of subsets of interacting genes determine which enzymes are produced, thus determining the rate at which metabolic reactions proceed. These reaction rates could then be viewed as constituting the next level of phenotypes. These in turn determine even higher level phenotypes, ultimately culminating in macroscopically observable ones where the concept of phenotype was originally introduced. In summary, any mapping from the states of an underlying collection of molecules to a higher level collective property of those molecules that may result from their interaction can be viewed as a generalization of the genotype–phenotype map, where the original conception of the latter corresponds to the special case where (i) the genes alone are sufficient to determine the higher level collective property and (ii) that higher level collective property is observable at the whole-organism level.

A more realistic basis upon which to build phenotypes than this outline of the historical trajectory contains is one that is not limited to genes alone, but includes all entities constituting a biological network. A phenotype must be a function of the levels of, for example, all of the molecular constituents that comprise it over time, even if more information is required to fully specify it. The aforementioned coarse-grained levels of biological network variables can thus be viewed as collectively determining the lowest level in a hierarchy of abstract phenotypes. In what proceeds, we will assume that we have a finite set *L* of variables and a finite set *P* of coarse-grained levels of each of those variables. These levels may have different units, but they can all be mapped into unitless quantities that account for the relevant scale of each variable. In general, each variable could take values in a distinct set *P*_*i*_, *i* ∈ *I* ranging over the variables, whereby *P* would be required to represent 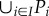 rather than a monolithic valuation set lacking any underlying substucture with respect to the variables under consideration. Then a possible state of our biological network is represented by a function *e*: *L* → *P* and coarse-graining a stationary distribution will lead to a probability distribution on the set of all maps, denoted *P^L^*, from subnetworks represented by subsets of *L* to the respective states of the variables that comprise them. We will refer to this more fine-grained generalization of the genotype–phenotype map, where arbitrary biological networks are substituted for genes and arbitrary networks states are substituted for phenotypes, as network–network state maps.

## 4. Probability distributions over network modules

Here, we describe examples of probability distributions over network modules. A more general presentation is provided in electronic supplementary material, §S3. As explained in §2, for a given biological subnetwork, the hypergraph representing the dependencies in the network context consists of subsets, *O*, of the variables, *L*, in the subnetwork. If we consider the case in which we have two variables *L* = {*l*_1_, *l*_2_} and there are two values, *P* = {0, 1}, then there are four possible assignments of values to variables each of which constitutes a state of the system. We will write the probability of each of these states as 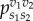 indicating that variable *v*_1_ is assigned value *s*_1_ and variable *v*_2_ is assigned value *s*_2_. A probability distribution over the states of the system for *L* is then given by4.1
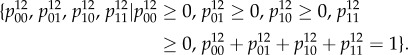
This imposes the standard conditions that probabilities are positive and sum to one. If we have the subset of *L* given by *O* = {*l*_1_}, then a probability distribution over its states is given by4.2
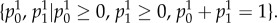
In order to be consistent, the distribution expressed in equation (4.1) should be related to that of equation (4.2) via a marginalization matrix4.3
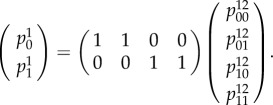


## 5. Compatibility of distributions on network–network state maps

Here, we provide an example of compatibility conditions on network–network state maps. A more general mathematical characterization of these constraints is provided in electronic supplementary material, §S5. When one has a non-trivial network architecture (corresponding to the SH hypergraph like those in figure 1), there will typically be more than one way of obtaining a probability distribution on a set by marginalizing a distribution on a larger set. For instance, if we have a network with three binary variables and two edges, {*l*_1_, *l*_2_} and {*l*_1_, *l*_3_}, then we can obtain a probability distribution on the set {*l*_1_} either by marginalizing probabilities defined over {*l*_1_, *l*_2_} as was done above or by marginalizing probabilities defined over {*l*_1_, *l*_3_} to obtain5.1
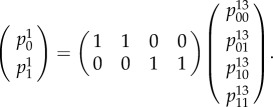
For an arbitrary choice of the quantities 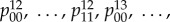 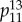, there is no reason that these two procedures should yield the same answers for 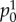 and 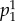. If one requires that they do yield the same answer, then one must impose consistency conditions. In our example, these conditions are as follows:5.2
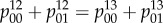
and5.3
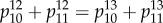


More generally, given a hypergraph 𝒢, we will be interested in two types of consistency conditions. We will say that a collection of probabilities associated to a hypergraph is *locally consistent* if, whenever two hyperedges share a subset in common, the probabilities for that subset obtained by marginalizing the probabilities associated to one of the hyperedges will agree with those obtained by marginalizing the probabilities associated to the other hyperedge. In our example above, there were only two hyperedges present, so the conditions we exhibited constitute the entirety of the local consistency conditions for that hypergraph. We will denote the set of all locally consistent probability distribution associated to a hypergraph 𝒢 as 𝕃(𝒢).

We will say that a collection of probabilities associated to a hypergraph is *globally consistent* if there exists a joint probability distribution on the totality of variables associated to the hypergraph such that the probabilities associated to any hyperedge are marginals of that joint distribution. In terms of our example, that would mean that there exist probabilities 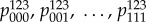 such that the following conditions hold:5.4
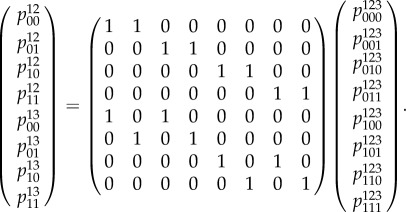
We will denote the set of all globally consistent probability distribution associated to a hypergraph 𝒢 as 𝕄(𝒢).

Because marginalizing from a set of random variables to a smaller set of variables can be accomplished by first marginalizing to an intermediate set and then marginalizing from the intermediate set down to the smaller set, it follows that global consistency implies local consistency. We will now see what conditions are needed in addition to local consistency to ensure global consistency.

As in our example, we can express marginalization from the set *L* of all variables down to a hypergraph 𝒢 in the form *v* = *Gx*, where *x* is a vector whose components are probabilities associated to *L*, *v* is a vector whose components are probabilities associated to 𝒢, and *G* is a suitable matrix. The consistency conditions can be expressed in terms of the fundamental spaces (kernel and cokernel) associated to this matrix [27]. In order for a vector *v* to be expressible as *Gx* for some *x*, we must satisfy the condition that *v* · *u* = 0 for all *u* ∈ coker(*G*). In our example, the cokernel of the matrix is spanned by the following two row vectors:5.5
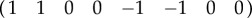
and5.6
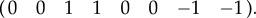
This leads to the conditions5.7
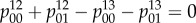
and5.8
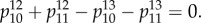
Note that these are precisely the local consistency conditions which we exhibited earlier. It can be shown that the condition that *u* · *v* = 0 for all *u* ∈ coker(*G*) will always be exactly the local consistency conditions (electronic supplementary material, §5).

To obtain the global consistency conditions, we note that, if *v* = *Gx*, then we also have *v* = *Gy* for any vector *y* such that *x*−*y* lies in the kernel of *G*. Choose a subspace *T* of column vectors which is transverse to ker(*G*) such that the union of *T* and ker(*G*) span the column space. Then the equation *v* = *Gx* has a unique solution if we restrict *x* to lie in *T*. In order for a column vector to represent a legitimate probability distribution, its components must all be non-negative. Hence, we conclude that *v* being globally consistent is equivalent to the following system of equations and inequalities having a solution:5.9
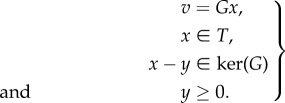


By using a method, such as Fourier–Motzkin elimination, to remove redundant inequalities, one can eliminate the quantities *x* and *y* from this system to obtain inequalities involving only the components of *v*. These are the global consistency conditions.

In our example, ker(*G*) is spanned by the following two column vectors:5.10
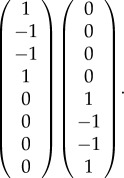
As our transverse space *T*, we will choose the space spanned by the following basis:5.11
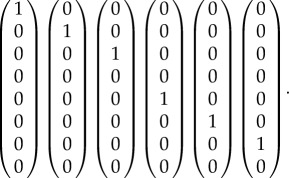
With this choice, the condition *x* ∈ *T* reduces to the equations *x*_4_ = *x*_8_ = 0. The conditions *x* − *y* ∈ ker(*G*) then become5.12
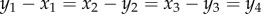
and5.13
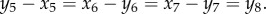
If we solve these for the *x*'s, substitute the result into the equation *v* = *Gx* and eliminate the *y*'s between the resulting equations and the inequalities *y* ≥ 0, we find the conditions *v* ≥ 0. This, of course, is just the condition that the probabilities be positive. Thus, for the case of this simple hypergraph, local consistency suffices to ensure global consistency. In §6, we will see that this is not always the case and that the inequalities obtained by elimination impose more conditions on the probabilities than just positivity.

## 6. Example of unsatisfiable constraints

We will now exemplify equations and inequalities that need to be satisfied in order to guarantee the consistency conditions for the case of three variables that form the simplest non-trivial cycle where inconsistency may arise. Suppose that *L* = {*l*_1_, *l*_2_, *l*_3_}, *P* = {0, 1}, 𝒢 = {{*l*_1_, *l*_2_}, {*l*_2_, *l*_3_}, {*l*_3_, *l*_1_}}.

**Figure 3.**
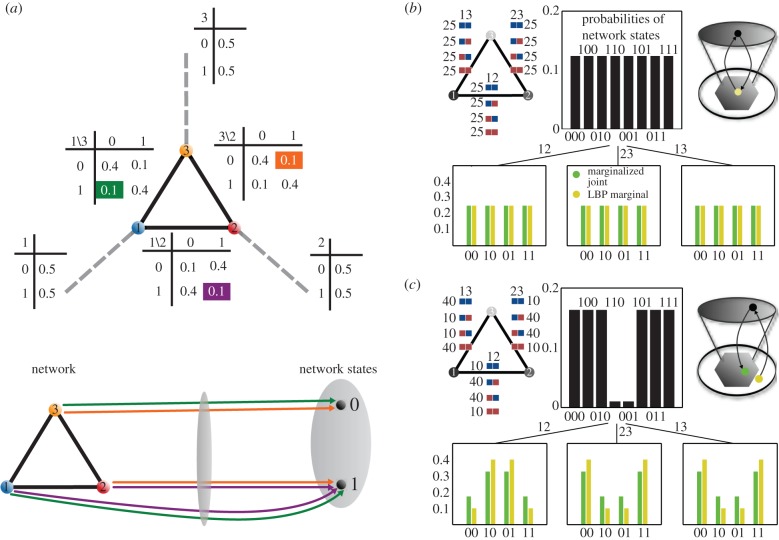
Model of inconsistent network state data. (*a*) An example structured according to the bottom row of figure 2*c*. The graph contains three nodes each representing one of the variables depicted in figure 2*a*. The dashed grey line coming from each variable points to the single variable marginal distribution depicted in the associated table. The pairwise edge marginal distributions are placed along the edges. The highlighted table entries (top) represent the constraint probabilities on the network–network state maps represented by the equivalently coloured arrows (bottom). The binary values representing variable states derive from the coarse-graining process over continuous network state data depicted in figure 2*b*. (*b*) (top-left) Representation of 300 samples comprising a dataset consistent with a uniform distribution over all network–network state maps from the model in (*a*). (top-middle) The joint probability distribution given in the top-left panel. The green bars in the bottom three panels represent the marginalization of this joint distribution according to the structure of the graph. The yellow bars in the bottom three panels represent the ostensible marginal distributions determined via the sum-product algorithm (loopy belief propagation) [28]. (top-right) A schematic where the top grey ellipse represents the space of joint probability distributions on three variables and the hexagon represents the pairwise marginals within their natural embedding space (figure 4). For this data, maximum-likelihood estimation (exact) and loopy belief propagation (approximate) yield equivalent points within the space of pairwise marginals. (*c*) Same as (*b*), but with data consistent with figure 2*c* bottom, which in the limit of a large amount of data would converge to the ostensible node and edge marginal distributions in (*a*). For the given dataset, maximum-likelihood estimation and loopy belief propagation yield different points within the natural embedding space of the pairwise marginals.

Local consistency means that the probability for the variable *l*_1_ to be associated to a given state is equivalent in case we marginalize over all the other variables contained in the biological network modules of which *l*_1_ is a component. Mathematically, this reduces to two equations corresponding to the cases when the state of *l*_1_ is 0 or 1. If we do likewise with *l*_2_ and *l*_3_ in place of *l*_1_, we obtain the set of local consistency conditions:6.1
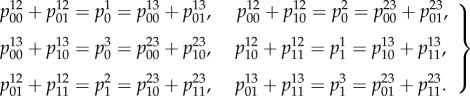
These result from applying the method outlined in §5 to enumerate all local consistency conditions. Using the local consistency conditions for our example, we can derive a set of inequalities that determine 𝕃(𝒢),6.2
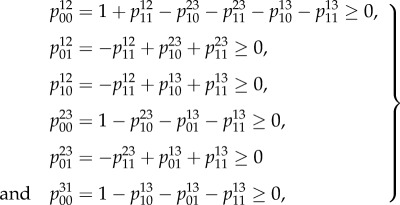
combined with the trivial inequalities that force all probabilities to be non-negative. Substituting the numbers from figure 3*a* (which are 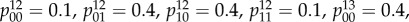 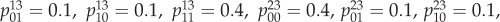 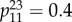) into equation (6.2), demonstrates that the local conditions are satisfied.

The global consistency conditions form an underdetermined system of linear equations for the putative global distribution so their solution will assume the form of a linear subspace. The following equations arise as a result of eliminating *x* from the equations determined by the conditions *v* = *Gx*, *x* ∈ *T*, 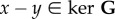:6.3
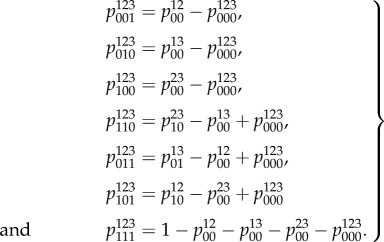
The remaining condition *y* ≥ 0 from equation (5.9) states that all the probabilities 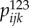 must be positive numbers, which is only possible if the putative marginals satisfy suitable inequalities given by6.4
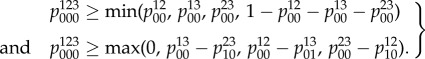


A minimal set of inequalities is then expressed by substituting the equalities from equation (6.2) into the inequalities determined by equation (6.4) and eliminating redundancies resulting in6.5
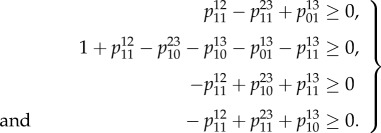
The inequalities from equation (6.2) and equation (6.5) combined with the non-negativity inequalities together determine the global polytype 𝕄(𝒢). For the example given in figure 3*a*, the first of the inequalities in equation (6.5) is demonstrated to be unsatisfied in equation (6.6)6.6
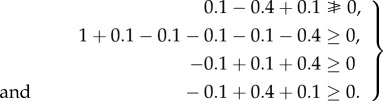


This indicates that data consistent with figure 3*a* could not derive from the network depicted there.

## 7. Cyclic network contexts can impose unsatisfiable constraints

Each node of the SH graph in figure 3*a* can be associated to the probability distribution that specifies probabilities for each biological variable to be observed in each of the states determined by the coarse-graining process described in §3. Each edge of the graph specifies a joint probability distribution for both of the nodes it contains (or connects) to simultaneously take on a given pair of values. Note that this does not imply the existence or absence of a physical interaction between the variables represented by these two nodes. Together, these probabilities represent constraints that the network context may impose upon the network. We assume three variables are observed via all possible pairwise combinations and that via the coarse-graining process we have binned the state of each variable into one of two classes. Each node of the graph in figure 3*a* represents a probability distribution over the observation of each variable in either of the two states established in the coarse-graining process. Each of the probability tables adjacent to each edge in the graph assigns a probability distribution to the set of maps from the nodes connected by the edge to all possible combinations of the network states. As these maps take collections of biological network variables as input and produce collections of network states as outputs, we refer to them as network–network state maps and thus to the associated probability distributions as probability distributions over network–network state maps.

Suppose the normalized contingency tables in figure 3*a* are meant to represent the ostensible structure and parameters of a biological process. It is often necessary to attempt to infer the parameters of such a model from data under the assumption that the structure of a given network architecture falls within the model class defined by a given graph. Figure 3*b* represents a case in which a hypothetical dataset is consistent with its derivation from a joint probability distribution, whereas figure 3*c* represents a case of inconsistency where the pairwise distributions are each individually consistent distributions, but, together, the three pairwise distributions are not consistent with any joint distribution over the states of all three network variables. This inconsistency is made possible by the fact that the network architecture in figure 3*a* contains a cycle [29–31] and that we have given an ostensible dataset leading to the inference of parameters that could not possibly derive from a joint probability distribution over all three network variables.

If this situation arises, it indicates some systematic error in the transfer of information whether it occurs intrinsically to the system, wherein a network has inconsistent constraints placed upon it by its network context or as part of the scientific data collection process. In the former case, this can be resolved by modifying the inconsistent constraints in such a manner that they become consistent with or without modifying the network architecture in doing so. In the latter case, this may result from employing a model which (i) takes insufficient account of the network context and (ii) relies on coarse-grained observations. In either case, the synthetic gene circuit schematized in electronic supplementary material, figure S4, serves as one mechanism implementing the example presented in electronic supplementary material, §S5.1. It consists of four genes each of which is capable of taking on three different states [32]. However, observing two out of the three states measured pairwise from three out of the four genes could result in data that would appear to be inconsistent. Such an observation would demonstrate without having to have knowledge of the correct network architecture, that the current model is insufficient to represent the underlying process.

For the case of the architecture in figure 3*a*, and moreover for any network architecture of any size that contains one or more cycles, the possibility of finding a joint distribution over all network variables that satisfies all constraints capable of being imposed upon it requires the implicit assumption that the structure of the network context can be viewed simultaneously as that of figure 2*c* top and that of figure 2*c* bottom. The spaces of probability distributions corresponding to the constraints that can be imposed upon the two network architectures contrasted in figure 2*c* are different. We can now apply the process described in §5 to classify the geometries and thus relationships among the spaces of probability distributions associated to constraints that can be imposed on all possible network architectures with a given number of variables.

## 8. Geometry of probabilistic constraints on network states

**Figure 4.**
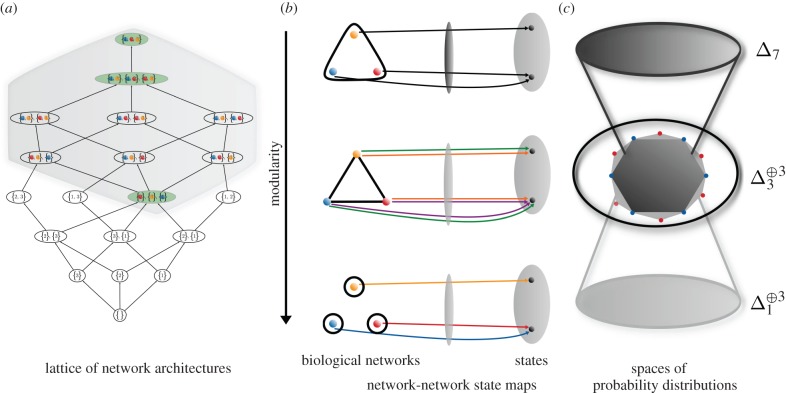
Relationship between biological network models and spaces of probability distributions. (*a*) The collection of all possible network architectures over three variables forms a lattice represented here by its Hasse diagram. An analogous lattice of network architectures exists for any number of variables. The Hasse diagram shows the manner in which network architectures are hierarchically related and are thus able to be embedded within one another. (*b*) Explicit examples of network–network state maps over three network architectures from (*a*) highlighted in green are represented as arrows mapping the variables represented as nodes of the graph underlying the network architecture into the collection of network state values determined by the coarse-graining chosen in figure 2*b*. There is a different collection of possible network–network state maps depending upon the structure of the network architecture. (*c*) Each collection of network–network state maps, one representative for each network architecture depicted in (*b*), is associated to a space of probability distributions defined over it. Moreover, the spaces of probability distributions associated to each graph are related via marginalization maps. The top level represents a joint probability distribution (i.e. Δ_7_: the eight-dimensional probability simplex) which can be marginalized to the middle space (i.e. 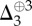: the union of three copies of the four-dimensional probability simplex) which in turn can be marginalized to the bottom space (i.e. 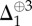: the union of three copies of the two-dimensional probability simplex). The light grey polytope in the middle, 𝕃(𝒢), represents the space of distributions consistent with the marginalization map from the middle to the bottom. The dark grey polytope, 𝕄(𝒢), represents the space of probability distributions consistent with marginalization from the top to the middle.

The relationships among possible network architectures are given by the lattice, which in this case indicates ordering by subset inclusion, of reduced subsets of biological network variables (i.e. collections of subsets of variables where no subset in the collection is a subset of another one, §2 and electronic supplementary material, §S2). For example, figure 4*a* shows the lattice of reduced subsets of three variables. We are only interested in those subsets that contain at least one instance of each variable. Restricting to the subsets of variables satisfying this condition corresponds to the region highlighted with a grey background in figure 4*a*. Each network architecture corresponds to a different modularization of the network–network state maps by the network context. For example, figure 4*b* shows in the same vertical order the different maps induced by the three architectures highlighted in green in figure 4*a*.

We consider those network architectures found lower in the lattice of figure 4*a* to be of higher modularity because each corresponds to the increasing restriction from placing constraints on higher- to placing constraints on lower- order correlations among variables. Figure 4*b* top corresponds to the least modular network architecture because constraints are placed upon correlations among all three variables. Figure 4*b* middle exhibits an elevated degree of modularity because constraints are placed upon correlations among pairs of variables. Similarly, figure 4*b* bottom is even more modular because constraints are placed upon each variable individually.

Each of the network architectures in figure 4*a* can be associated to a pair of spaces of probability distributions over network–network state maps. These correspond to the spaces of globally, 𝕄(𝒢), and locally, 𝕃(𝒢), consistent distributions described in §5 and electronic supplementary material, §S5. Figure 4*c* schematically depicts the relationships among the probability distributions associated to the corresponding architectures and network–network state maps in figure 4*b*. For figure 4*c*(i), 𝕄(𝒢) = 𝕃(𝒢). The inconsistency noted in the previous section between the architectures figure 4*b*(i) and (ii) is a result of the differing geometries in figure 4*c*(ii). There, the smaller darker grey region, 𝕄(𝒢), defined by the inequalities expressed in equations (6.2) and (6.5) corresponds to the space of probability distributions defined over all possible network–network state maps associated to the network architecture in figure 4*b*(ii). Similarly, the lighter grey region defined by equation (6.3) alone corresponds to 𝕃(𝒢) for figure 4*b*(ii) and thus 𝕄(𝒢) < 𝕃(𝒢) in the latter case.

## 9. Naive likelihood of sampling unsatisfiable constraints

Relationships between spaces of potential constraints placed upon patterns of network states like that of figure 4*c*(ii) occur for all network architectures defined over any number of variables so long as there exists at least one cycle in the corresponding network architecture, §7. For the case of three variables, there is only one class of graphs containing a cycle, which is that of figure 4*b*(ii). For the case of four variables, there are nine different classes of hypergraphs containing cycles and these nine classes can be split into two groups depending upon whether or not the edges of the graphs are each restricted to represent correlations among only two variables. Electronic supplementary material, figure S5, shows the components of the analogous lattice to that of figure 4*a* as well as these different classes of network architectures on four variables having cycles.

Given this larger collection of network architectures with cycles, we can assess the relative sizes of the spaces 𝕄(𝒢) and 𝕃(𝒢) (figure 4*c*(ii)) of probability distributions over network–network state maps. We assess the likelihood of choosing a point in 𝕄(𝒢) at random by computing the ratio of the volume of 𝕄(𝒢) (associated to the non-modular network architectures analogous to that of figure 4*b*(i) with a single edge containing all four variables), whose architecture and thus volume is fixed, to that of 𝕃(𝒢), whose volume varies according to each of the cyclic graphs associated to a network architecture on four variables. We refer to this number as the global : local volume ratio or Vol(𝕄(𝒢))/Vol(𝕃(𝒢)) (see §5 and electronic supplementary material, §§S5 and S6). The comparison defined by this ratio is meaningful since 𝕃(𝒢) (electronic supplementary material, equation S23) and 𝕄(𝒢) (electronic supplementary material, equation S24) are of the same dimension. In the case where the constraints defining 𝕃(𝒢) are eliminated, the analogue of this volume ratio would be 0 for all 𝒢. This volume ratio determines the *a priori* likelihood of observing inconsistency for a given network architecture. The consistency check involved in computing this ratio can be used as a test demonstrating, for those cases exhibiting inconsistency, that the model being used is incorrect in the sense that it does not correspond sufficiently to the actual network context determining the constraints placed upon the network. Consider the probability of locally versus globally consistent *observations* (*p*(𝕃(𝒢)_O_) versus *p*(𝕄(𝒢)_O_), respectively) separately from the probability of locally versus globally consistent *models* (*p*(𝕃(𝒢)_M_) versus *p*(𝕄(𝒢)_M_), respectively) that accurately reflect the underlying process. We can then estimate the probability of having a locally consistent model despite obtaining globally consistent observations, *p*(𝕃(𝒢)_M_|𝕄(𝒢)_O_), via a simple application of Bayes' theorem
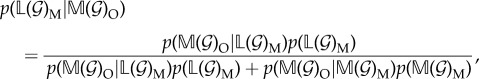
where *p*(𝕄(𝒢)_O_|𝕄(𝒢)_M_) = 1, the volume ratio described above corresponds to *p*(𝕄(𝒢)_O_|𝕃(𝒢)_M_), and one could consider the impact of different prior probabilities, *p*(𝕃(𝒢)_M_), of having a locally consistent model.

**Figure 5.**
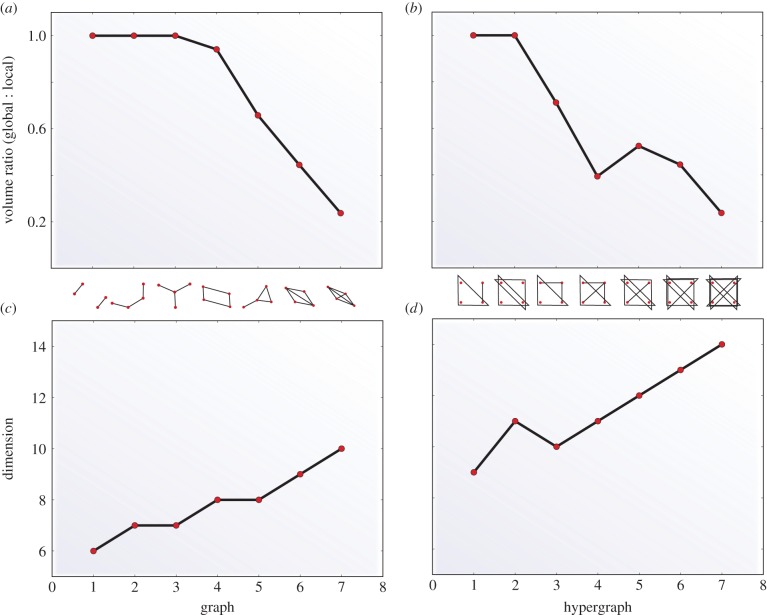
Non-modular to modular probability space volume ratio. (*a*,*b*) Ratio Vol(𝕄(𝒢))/Vol(𝕃(𝒢)) associated to 2-regular and non-2-regular network architectures, respectively. The (hyper)graph associated to each value of the volume ratio is displayed along the *x*-axis of each panel. (*c*,*d*) Natural dimension of the space of probability distributions associated to 𝕄(𝒢) and 𝕃(𝒢) for each hypergraph.

Figure 5*a*,*b* shows the results of computations of this global : local volume ratio for 14 different hypergraphs. Figure 5*c*,*d* shows the dimension of the spaces within which these volumes are computed. The spaces are equivalent and thus the volume ratio equal to one for graphs lacking cycles (e.g. the first three graphs along the *x*-axis of figure 5*a*). For the nine network architectures in figure 5*a*,*b* containing cycles, the volume ratio is strictly less than one. This quantifies the probability that the network architecture depicted along the *x*-axis will be able to satisfy the constraints that the associated network context is capable of placing upon it.

## 10. Potential for unsatisfiable constraints may bias the sampling of network architectures by evolutionary processes

**Figure 6.**
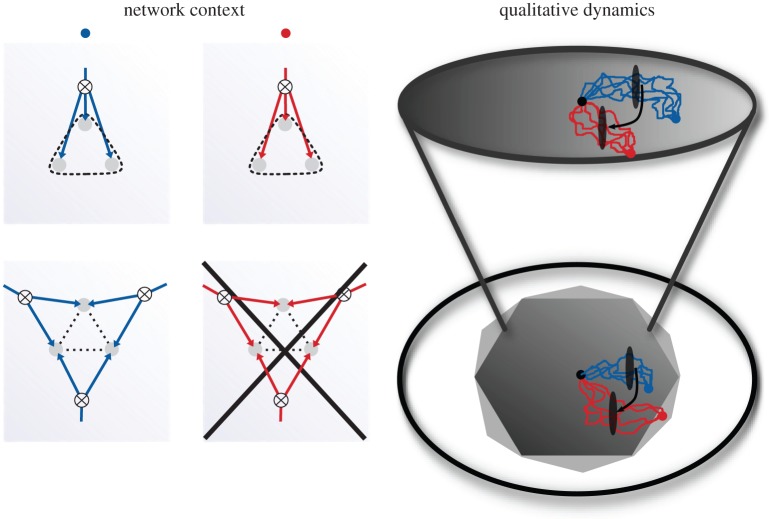
Constraints imposed on stochastic biological networks and evolutionary dynamics by network architecture. Schematic of a potential network context (left) for each of the hypothetical stationary probability distributions associated to the fitness peak established by the blue and red points within the spaces of probability distributions represented on the right. Either of the two network architectures represented on the left are capable of achieving the stationary distribution over network–network state maps specified by the blue stationary distribution associated to a hypothetical fitness peak. On the other hand, only the network architecture from the top (and not the bottom) is capable of achieving the red stationary distribution representing an alternative potential fitness peak.

The satisfiability of constraints capable of being placed on the various architectures is logically a function of whether or not the network architecture is cyclic or acyclic. For those network architectures containing cycles, there are certain functional requirements that can be achieved so long as only local and not global consistency is required of them. Once global consistency is imposed as in the structure corresponding to the joint correlations among all variables, those functions that were accessible when only local consistency was imposed are unavailable. For acyclic network architectures, there is no difference between the satisfiability of locally or globally imposed constraints. Figure 6 right shows a schematic of one potential scenario by which a given cyclic network architecture may be selected against. The black points in the centre represent an initial condition of a stochastic process that is selected for its ability to achieve one of two different stationary distributions represented by the blue and the red points, respectively. This is equivalent to placing a fitness landscape given by a function whose maximum is located at the given points and defined over the relevant space of probability distributions. The network architecture represented in the top row of figure 6 is able to achieve as its stationary distribution any of the constraints capable of being imposed upon it that are consistent with its architecture because it is acyclic. On the other hand, the network architecture in the bottom row is incapable of achieving certain constraints that may be imposed upon it by a network context consistent with its architecture because it is cyclic.

When selective pressure is induced equivalent to the distribution located at the blue point, or at any other point within the dark grey region, either of the architectures are essentially equivalent with respect to the statistics of samples from their corresponding probability distributions and they can thus be considered as members of an evolutionarily neutral space. On the other hand, selective pressure equivalent to the probability distributions located at the red point differentiates between the networks of the top and bottom row or equivalently between the network of the bottom row when global consistency is imposed versus the same network when only local consistency conditions are imposed. The same qualitative relationship holds true for the spaces of probability distributions of all network architectures of any size and for any number of different levels in the discrete coarse-graining of network states so long as the graph associated to the relevant correlations among variables contains at least one cycle.

The distinction between cyclic and acyclic network architectures with respect to the ability to have unsatisfiable constraints placed upon them is sharp. However, within the class of cyclic network architectures, the likelihood of having unsatisfiable constraints imposed on a given network architecture increases, at least approximately, with the number of cycles in the given network architecture (figure 5 and §9). This indicates that the strength of selection against network architectures with a larger number of nested cycles is likely to be stronger than that against network architectures with a relatively smaller number of cycles. Initiating an evolutionary process with a large network containing many nested cycles may then result in the elimination of some via any process that can result in cycle breakage until the number of nested cycles decreases sufficiently so that the intrinsic strength of selection against cycles reaches equilibrium with the rate at which new cycles form. One possibility, depending upon the overall relationship between these rates, is a hierarchical-modular one where a globally hierarchical network has a number of cyclic modules, each of whose size is small relative to the overall size of the network, interspersed throughout.

## 11. Discussion

When biological networks are studied, we remove a subnetwork from a larger context [33]. Depending upon the scale of the study, the boundary between subnetwork and network context may vary. For example, in a relatively small-scale study, the subnetwork may consist of a few genes and metabolites where the context comprises other genes, metabolites and intracellular structures. For relatively large-scale models attempting to take into account all of the processes comprising a single-celled organism, the network context consists of the variables in that organism's environment. In even larger scale studies of multicellular organisms, populations or communities, the same general principle applies by appropriately shifting the boundary between the subnetwork and network context.

One salient feature applying at any scale is that the structure of the network context plays a crucial role in determining whether or not unsatisfiable constraints on the stochastic dynamical patterns of network states may arise at all. We note based on previously existing results that mutually incompatible constraints are only capable of arising when the network architecture contains a cycle. Moreover, our results suggest the likelihood of mutually incompatible constraints arising relative to network architecture increases with the number of cycles in that network architecture. An evolutionary process exhibiting uniform sampling over the space of network architectures and the space of possible constraints within each network architecture would thus be expected to exhibit a bias towards the breakage of cycles. One would not expect such a bias to eliminate the existence of cycles in biological networks. However, it is reasonable to expect on the basis of this result a kind of hierarchical modularity: where modules that may possess cycles and are small relative to the overall size of the network exist within a globally hierarchical network structure. Of course, there are other factors which may contribute to the development of such network architectures.

It will be important in future work to examine this prediction more closely in the context of developing bottom-up stochastic process models that allow for the explicit encoding and solution of models of more complex biological networks [34,35]. It is possible that the specific dynamics of a given network context may lead to apparent access to correlations that are otherwise inaccessible. In the case of gene-regulatory networks, this may occur via a form of *cis*-regulation that enables the breakage of statistical dependence in a time-dependent manner (electronic supplementary material, figure S4). But such a scenario seems much less plausible than the ability to resolve inconsistency by breaking cycles in the network architecture. In the long term, the latter corresponds to what is observed in hierarchically organized transcription factor networks [21,36–38]. The mechanism outlined here is consistent with previous analyses of hierarchical-modular gene-regulatory network architectures [36–42].

To contribute to the broader goal of establishing an integrated framework that synthesizes hypothesized intrinsic and extrinsic constraints necessary to understand the functioning and evolution of biological systems, here we have traced a path from biological network architecture to network state constraint satisfiability, and, via the impact of network states on higher level properties culminating in macroscopically observable phenotypes, to evolutionary processes. In the particular context of gene-regulatory networks, one goal of measuring gene expression at transcriptomic scale is to uncover the structure of the generative process encoded in the interactions involved, but, so far, even the most sophisticated methods of describing them at the mechanistic level are only solvable for extremely simple regulatory network architectures [34,35]. This fact has, in part, motivated computational biologists to develop a large collection of algorithms to infer aspects of this structure [1,43] and experimental biologists to compare networks on the basis of their hierarchical and modular architecture [44]. Our model and its framework put forward a class of fundamental constraints that may impact the expected structure of biological networks. The fact that the satisfiability of the space of possible constraints that can be imposed upon a network is dependent upon the structure of the network context provides a mechanism by which natural selection may exhibit a fundamental bias in its sampling of biological network architectures.

## Supplementary Material

Supplementary Material
