# Supplementary material for: Potential unsatisfiability of cyclic constraints on stochastic biological networks biases selection towards hierarchical architectures
Source: J R Soc Interface. 2015 Jul 6;12(108):20150179. doi: 10.1098/rsif.2015.0179 (PMC4528583; doi:10.1098/rsif.2015.0179)
Supplement: Supplementary Material [file rsif20150179supp1.pdf]

Supplementary Material

S1 Outline

In the Supplementary Material we provide a more formal mathematical description of the results we make use of in the main text. In Sec. S2 we characterize biological network architectures as a collection of subsets, each individually referred to as a module, of network variables that defines a hypergraph over those network variables. Sec. S3 provides a functorial description of probability distributions defined over such network architectures and the mappings between those network architectures and the states of the modules of the network. Sec. S4 characterizes the manner in which a hierarchy of coarse-grained network states can be viewed as a refinement of the genotype-phenotype map, where the genotype and phenotype correspond to two different levels within this hierarchy, but maps between any two levels are considered to define valid coarse-grainings. Sec. S5 provides a sheaf theoretic formulation of the local and global consistency conditions that are logically imposed upon probability distributions over collections of such maps from some lower- to some higher-level in the hierarchy of coarse-grainings. Sec. S6 complements Sec. 5 of the main text providing a detailed example computation of the ratio of volumes between the polytopes corresponding to the global and local consistency conditions for the four-cycle network architecture.

S2 Biological network architecture

A module of a biological network is represented by a subset of variables, $O \subseteq L$. A biological network architecture, $\mathcal{G}$, may then be represented by a subset of all possible such modules. This is to say that $\mathcal{G}$ is a subset of the set of all subsets of L, $\mathcal{G} \subseteq \mathcal{P}(L)$, that satisfies the following two conditions

1. $\cup_i O_i = \cup \mathcal{G} = L$,
2. If $O, O' \in \mathcal{G}$ and $O \subseteq O'$ then $O = O'$.

The first condition is just a statement that $\mathcal{G}$ represents a decomposition of the collection of all variables under consideration into subsets and this is why we refer to $\mathcal{G}$ as a collection of biological network modules. The second condition means simply that we will not consider nested subsets and so we will take for our $O \in \mathcal{G}$ the biggest $O \in \mathcal{G}$ that is not a subset of some other $O' \in \mathcal{G}$. The second condition also implies that if a given subset of variables O' is compatible in a sense to be explained more precisely in what proceeds then any smaller subset of variables O is also compatible.

Mathematically, the two conditions given above state that $\mathcal{G}$ is a *covering* of the set L. This is equivalent to $(L, \mathcal{G})$ being a reduced hypergraph, Sperner family, or clutter over L [28]. Coverings $\mathcal{G}$ of the space of biological network variables contain the necessary information to make precise what we heuristically refer to at other points in this paper as modularity in order to cohere with standard terminology in systems biology literature while attempting to submit our own precise interpretation of the relatively colloquial concept.

S3 Functorial formulation of probability distributions over network modules

As stated in Sec. 4, essentially all studies of biological networks consider states of subsets of variables that interact either directly or indirectly. We will represent these modules as subsets of L and their states as functions from these subsets to P.

The power set of L, which we shall denote as $\mathcal{P}(L)$, can be regarded as a category [45–48] in which the objects are subsets of L and morphisms represent inclusion of a smaller subset into a larger superset (i.e. $O \subseteq O' \Rightarrow O \to O'$).

Before proceeding, we define a few technical terms from the theory of sheaves and presheaves. We do not provide all necessary definitions to make use of the theory in more abstract contexts for which we direct the reader to [45]. Given L we define a **presheaf** over it to be a contravariant functor, $PSh\colon \mathcal{P}(L)^{opp} \to \mathbf{Set}$, from the category of subsets of L, $\mathcal{P}(L)$, to the category of sets, **Set**. Thus for every $U \in \mathcal{P}(L)$, $PSh(U)$ is a set.

$s \in PSh(U)$ is a **local section** over U with respect to PSh. A **covering** of L with respect to $\mathcal{P}(L)$ is an indexed set $\{O_i\}_{i\in I}$ where $O_i \subseteq L$ such that $\cup_{i\in I} O_i = L$. A **system of local sections** over a covering $\{O_i\}_{i\in I}$ is a set of ordered pairs of elements, O_i, of the covering and sections, $s_i \in PSh(O_i)$, that comprise a set of the form

$$\{(O_i, s_i) \mid i \in I,\ s_i \in PSh(O_i)\}.$$

A system of local sections is **globally consistent** when there exists $s \in PSh(L)$ such that for all $i \in I$

$$[PSh(O_i \to L)](s) = s_i,$$

where, s is called a **witness** to global consistency. A system of local sections is said to be **locally compatible** when for all $i, j \in I$ the following are satisfied

$$[PSh(O_{ij} \to O_i)](s_i) = [PSh(O_{ij} \to O_j)](s_j),$$

where $O_{ij} = O_i \cap O_j$. Note that if a system of local sections is globally consistent then it is locally compatible. A presheaf is said to be a **sheaf** when given any locally compatible system of local sections, the system of local sections is both globablly consistent and there exists a unique witness. We refer to a presheaf that satisfies the existence but not the uniqueness condition as a **half-sheaf**.

The sheaf condition is also commonly expressed in terms of an equalizer diagram [46]. PSh is a sheaf if beginning with the lattice of inclusions among subsets of network variables

$$L \overset{\rho}{\dashleftarrow} \coprod_{i\in I} O_i \underset{\rho_j}{\overset{\rho_i}{\leftleftarrows}} \coprod_{i,j\in I\times I} O_{ij}\ , \tag{S1}$$

for any covering $\{O_i\}_{i\in I}$ and applying the PSh functor to Eq. S1 results in

$$PSh(L) \overset{PSh(\rho)}{\dashrightarrow} \coprod_{i\in I} PSh(O_i) \underset{PSh(\rho_j)}{\overset{PSh(\rho_i)}{\rightrightarrows}} \coprod_{i,j\in I\times I} PSh(O_{ij})\ , \tag{S2}$$

where there exists $s \in PSh(L)$, such that all of the following conditions are satisfied

1. $[PSh(\rho)](s) = \{s|_{O_i} \mid i \in I\}$,
2. for a family $s_i \in PSh(O_i)$: $[PSh(\rho_i)](s_i) = \{s_i|_{O_{ij}}\}$ and $[PSh(\rho_j)](s_i) = \{s_j|_{O_{ij}}\}$,
3. s is unique in satisfying conditions 1 and 2 among elements of $PSh(L)$.

In this notation, if condition 3 is not satisfied, then PSh is a half-sheaf.

Given a presheaf and an associated covering, we may ask when it is the case that every locally compatible system of local sections over the covering is globally consistent. If this is the case, the covering is said to be **half-sheaf-like** because for the presheaves we study there is, in general, more than one witness to global consistency.

None of the presheaves we work with in this paper are sheaves, except in degenerate cases. We work exclusively with presheaves and their coverings. Some coverings are half-sheaf-like. Surprisingly, some are not. This is to say that, if a covering is not half-sheaf-like, then not every locally compatible system of local sections over the covering is globally consistent [7, 48–51]. The latter correspond to network architectures containing cycles whereas the former are acyclic.

A state of a subset of variables, $O \subseteq L$, is an assignment of values in P to each variable in O which is a tuple of length $|O|$ containing elements from P. This correspondence is determined by the presheaf functor $\mathcal{E} = \mathrm{Hom}(-, P)$. Specifically, this functor may be described as

$$\begin{aligned} \mathcal{E} \colon \mathcal{P}(L)^{opp} &\to \mathbf{Set} \\ O &\mapsto P^O, \\ O \subseteq O' &\mapsto \{e' \mapsto (e' \circ \iota) \mid e' \in P^{O'}\}, \end{aligned} \tag{S3}$$

where $\iota \colon O \to O'$ is the injection of the subset O into O' (i.e. $\iota(o) = o$ for all $o \in O$). In this case, $\mathcal{E}$ is a sheaf, but note that this is not the case for the distribution presheaf, $\mathcal{D}$, considered later. For example, if we consider the case in which we have two variables $L = \{l_1, l_2\}$ and there are two potential states, $P = \{0, 1\}$, then $\mathcal{E}$ operates on the lattice of subsets generated by L to give spaces of functions containing the possible network-network state maps as exemplified in Fig. S1. For example, $\mathcal{E}(\{l_1, l_2\}) = \{e^{12}_{00}, e^{12}_{01}, e^{12}_{10}, e^{12}_{11}\}$ where $e^{12}_{01}(l_1) = 0$ and $e^{12}_{01}(l_2) = 1$. As another example, $\mathcal{E}(\{l_1\}) = \{e^1_0, e^1_1\}$ where $e^1_0(l_1) = 0$ and $e^1_1(l_1) = 1$. $\mathcal{E}(\{l_1\} \subseteq \{l_1, l_2\})$ is given explicitly by

$$\begin{aligned} e^{12}_{00} &\mapsto e^1_0 \\ e^{12}_{01} &\mapsto e^1_0 \\ e^{12}_{10} &\mapsto e^1_1 . \\ e^{12}_{11} &\mapsto e^1_1 \end{aligned} \tag{S4}$$

Next, we introduce extended probability distributions by defining a functor $\mathcal{D}$ that will compose with $\mathcal{E}$ to convert collections of network-network state maps into probability distributions over them. Given a finite set S, define $\mathcal{D}(S)$ to be the set of all maps from $\mathcal{P}(S)$ to the interval $[0, 1]$ which satisfy the following two conditions: For all $d \in \mathcal{D}(S)$, we have $d(S) \in \{0, 1\}$.[1] For all $d \in \mathcal{D}(S)$ and all $A, B \subset S$, we have $d(A) + d(B) = d(A \cup B) + d(A \cap B)$.

Returning to the running example,

$$\begin{aligned} \mathcal{D}(\mathcal{E}(\{l_1, l_2\})) &= \{p^{12}_{00}, p^{12}_{01}, p^{12}_{10}, p^{12}_{11} \mid p^{12}_{00} \geq 0, p^{12}_{01} \geq 0, p^{12}_{10} \geq 0, p^{12}_{11} \geq 0, p^{12}_{00} + p^{12}_{01} + p^{12}_{10} + p^{12}_{11} = 1\}, \\ \mathcal{D}(\mathcal{E}(\{l_1\})) &= \{p^1_0, p^1_1 \mid p^1_0 \geq 0, p^1_1 \geq 0, p^1_0 + p^1_1 = 1\}. \end{aligned} \tag{S5}$$

If S and S' are finite sets, which in our case will usually be sets of network-network state maps given by $\mathcal{E}(O)$, $d \in \mathcal{D}(S)$ and $d' \in \mathcal{D}(S')$ are probability distributions over these spaces, and $f \colon S \to S'$ is a partial function, we will say that f is compatible with d and d' when, for all $X \in \mathrm{img}(f)$, we have

$$d'(X) = \begin{cases} \frac{d'(\mathrm{img}(f))}{d(\mathrm{dom}(f))} d(f^{-1}(X)) & d(\mathrm{dom}(f)) \neq 0, \\ 0 & d(\mathrm{dom}(f)) = 0. \end{cases} \tag{S6}$$

In other words, the map f preserves ratios of probabilities of events. In the case where f is a partial surjection ($\mathrm{img}(f) = S'$), compatibility completely determines d' in terms of d and thus $\mathcal{D}$ may be regarded

[1] Normally, we would only have $d(S) = 1$, but since we want to introduce conditionalization in a coherent way it becomes necessary to admit degenerate distributions where $d(S) = 0$ as well. This simplifies the exposition by not requiring us to worry about dividing by zero and having to introduce special cases when dealing with conditional probabilities and partial functions. Of course, it also means that we cannot automatically assume that an element of $\mathcal{D}(S)$ can be normalized without checking this fact but in our examples, this verification will turn out to be routine and trivial.

as a functor from the subcategory of sets with partial surjections as morphisms to transformations on probability distributions:

$$d' = \mathcal{D}(f)(d) = \begin{cases} X \mapsto \frac{d(f^{-1}(X))}{d(\mathrm{dom}(f))} & d(\mathrm{dom}(f)) \neq 0, \\ X \mapsto 0 & d(\mathrm{dom}(f)) = 0. \end{cases} \tag{S7}$$

Specifically, when f is a total surjection, this map corresponds to marginalization. For example, in the case $f = \mathcal{E}(\{l_1\} \subseteq \{l_1, l_2\})$

$$\begin{aligned} \mathcal{D}\big(\mathcal{E}(\{l_1\} \subseteq \{l_1, l_2\})\big) \colon \mathcal{E}(\{l_1\} \subseteq \{l_1, l_2\}) \to \mathcal{E}(\{l_1\}), \\ d \mapsto d', \end{aligned} \tag{S8}$$

then

$$d'(\{e^1_0\}) = \mathcal{D}(f)(d)(\{e^1_0\}) = \frac{d(f^{-1}(\{e^1_0\}))}{d(\{e^{12}_{00}, e^{12}_{01}, e^{12}_{10}, e^{12}_{11}\})} = \frac{d(\{e^{12}_{00}, e^{12}_{01}\})}{1} = d(\{e^{12}_{00}\}) + d(\{e^{12}_{01}\}) = p^{12}_{00} + p^{12}_{01}. \tag{S9}$$

When f is a partial isomorphism, it corresponds to conditionalization. For example, if f is defined such that we condition on variable one being in state zero, $l_1 = 0$,

$$f \colon \{e^{12}_{00}, e^{12}_{01}\} \subset \mathcal{E}(\{l_1, l_2\}) \to \{f(e^{12}_{00}), f(e^{12}_{01})\} \tag{S10}$$

then

$$d'(\{f(e^{12}_{00})\}) = \mathcal{D}(f)(d)(\{f(e^{12}_{00})\}) = \frac{d(f^{-1}(\{f(e^{12}_{00})\}))}{d(\{e^{12}_{00}, e^{12}_{01}\})} = \frac{d(\{e^{12}_{00}\})}{d(\{e^{12}_{00}\}) + d(\{e^{12}_{01}\})} = \frac{p^{12}_{00}}{p^{12}_{00} + p^{12}_{01}}. \tag{S11}$$

Finally, when f is a general partial surjection, it corresponds to a combination of conditionalization and marginalization.

In order to admit the basic tools of linear algebra for the purpose of calculations regarding relationships between spaces of probability distributions we explain how they embed into linear spaces. By definition, an extended probability distribution $p \in \mathcal{D}(S)$ is an element of $\mathbb{R}^S$. We denote the inclusion map as

$$\mathrm{emb(S)} : \mathcal{D}(\mathrm{S}) \to \mathbb{R}^{\mathrm{S}}. \tag{S12}$$

Because a convex combination of two probability distributions is again a probability distribution, the image of $\mathrm{emb}(S)$ consists of a convex set and the origin point (corresponding to the degenerate zero distribution). Furthermore, if n is the number of elements of the set S, this convex set works out to be the probability simplex with n vertices, which we denote Δ_{n-1}. In our example above, $\mathcal{D}(\mathcal{E}(\{l_1, l_2\}))$ is the tetrahedron Δ_3. Since any vector $v \in \mathbb{R}^S$ may be written as $v = c_+ p_+ - c_- p_-$ where $c_+, c_- \in [0, \infty)$ and p_+ and p_- are probability distributions, the image of $\mathrm{emb}(S)$ spans the vector space $\mathbb{R}^S$. For purposes of later reference, note that, if $f \colon S \to S'$ is a partial surjection, then $\mathcal{D}$ extends to a fractional linear map, as in Eq. S11, from $\mathbb{R}^S$ to $\mathbb{R}^{S'}$ and that, in the special case where f is a total surjection, as in Eq. S9, it is in fact a linear map.

S4 Precise formulation of coarse-graining network states

As described in Sec. 3 it is also possible to consider network states that derive from coarse-graining lower-level network states. Once this is done, one arrives at probability distributions over network modules like that introduced in Sec. 4. As a result of this, our conclusions that are formulated in terms of a single level of coarse-graining network-network state maps also apply to coarse-graining over multiple levels at once despite the fact that the parameters of the relevant probabilistic model are likely to be different.

For each subset of variables $O \in \mathcal{P}(L)$, let $\phi_i(O)$ be the set of network states at level i, which can be determined from the expression levels of variables in O. Note that $\phi_i(O)$ may be empty if the set O does not contain enough variables to determine the values of any network state at level i. When $O_1 \subseteq O_2 \in \mathcal{P}(L)$, we have a restriction map $\pi_i^{O_2O_1} : \phi_i(O_2) \to \phi_i(O_1)$. These maps satisfy the consistency conditions that π_i^{OO} is the identity map and that $\pi_i^{O_3O_2} \circ \pi_i^{O_2O_1} = \pi_i^{O_3O_1}$, i.e. π_i is a functor on $(\mathcal{P}(L), \subseteq)$. As stated earlier, we set $\phi_1(O) = P^O$ and $\pi_1^{O_2O_1}$ to be the restriction map from P^{O_2} to P^{O_1}. If $i \leq j$, let $\Omega_{ij}(O) : \phi_i(O) \to \phi_j(O)$ be the coarse-graining map which describes how higher level network states are determined from lower level network states. These maps are all surjections and, for consistency, we will require the following conditions:

1. $\Omega_{ij}(O) \circ \Omega_{jk}(O) = \Omega_{ik}(O)$ whenever $i \leq j \leq k$.
2. $\Omega_{ii}(O)$ is the identity map on $\phi_i(O)$.
3. If $O_1 \subseteq O_2 \in \mathcal{P}(L)$ and $i > j$, then $\Omega_{ij}(O_1) \circ \pi_i^{O_2O_1} = \pi_j^{O_2O_1} \circ \Omega_{ij}(O_2)$

In other words, Ω must be suitably functorial in both of its arguments.

For example, if our lower level network states for a set of variables $O_1 = \{l_1, l_2, l_3, l_4\}$ are given by a set of binary sequences, then the projection of these network states down to the set $O_2 = \{l_3, l_4\}$ followed by mapping to the higher level network states $x = \{01, 10\}$ and $y = \{11\}$ is equivalent to first mapping to the higher-level network states X and Y and then projecting down to O_2 shown by the equivalent paths from the top-left to the bottom-right in Fig. S2A. Of course, there is an equivalent diagram for the subset $\{l_1, l_2, l_3\}$.

Since the map $\Omega_{1i}(O)$ is a surjection from P^O onto $\phi_i(O)$, we can use it to map our probabilistic structures to $\phi_i(O)$. Set $\mathcal{E}_i = \Omega_{1i}(O)^{-1} \circ \mathcal{E}$ and $\mathcal{D}_i = \Omega_{1i}(O)^{-1} \circ \mathcal{D}$. Then we end up with the overall relationships summarized in Fig. S3. As a consequence of the consistency conditions the coarse-graining maps ϕ and Ω, there is a natural transformation between the functors $\mathcal{E}_i$ and $\mathcal{E}_{i+1}$ implying that the following diagram commutes

$$\begin{array}{ccc} \mathcal{E}_{i+1}(O_1) & \xrightarrow{\mathcal{E}_{i+1}(\subseteq)} & \mathcal{E}_{i+1}(O_2) \\ \downarrow{\scriptstyle t_{O_1}} & & \downarrow{\scriptstyle t_{O_2}} \\ \mathcal{E}_i(O_1) & \xrightarrow{\mathcal{E}_i(\subseteq)} & \mathcal{E}_i(O_2) \end{array}$$

for any $O_2 \subseteq O_1$.

Given a covering $\mathcal{G}$ of the space of biological network variables, we can consider the higher order network states associated to the elements of $\mathcal{G}$. For a suitable choice of cover and a suitable level of network states, it may happen that the network states associated to different elements of $\mathcal{G}$ are distinct. For instance, in the example of Fig. S2, if we take $\mathcal{G} = \{O_1, O_2\}$ where $O_1 = \{l_1, l_2, l_3\}$ and $O_2 = \{l_3, l_4\}$, we have $\phi_{i+1}(O_1) = \{u, v\}$ and $\phi_{i+1}(O_2) = \{x, y\}$. In such a case, if we were to perform one experiment which measured the network states $\{u, v\}$ and another experiment which measured $\{x, y\}$, then the result could be understood as examining the covering $\{O_1, O_2\}$ at network state level $i + 1$.

S5 Sheaf-theoretic formulation of compatibility of distributions on network-network state maps

Given a covering of the space of variables $\mathcal{G}$, a compatible family for $\mathcal{G}$ with respect to $\mathcal{D} \circ \mathcal{E}$ is given by a family of distributions $\mathcal{D}(\mathcal{E}(\mathcal{G})) = \{d_O \in \mathcal{D}(\mathcal{E}(O)) | O \in \mathcal{G}\}$ such that for all $O, O' \in \mathcal{G}$

$$d_O | O \cap O' = d_{O'} | O \cap O'. \tag{S13}$$

This first set of conditions is later referred to as local consistency. The space of all such locally consistent distributions for a given covering, $\mathcal{G}$, is referred to as $\mathbb{L}(\mathcal{G})$ where

$$\mathbb{L}(\mathcal{G}) = \{d_O \in \mathcal{D}(\mathcal{E}(\mathcal{G})) \mid (\forall O, O' \in \mathcal{G})\ d_O|O \cap O' = d'_O|O \cap O'\}. \tag{S14}$$

These conditions mean that any two distributions d_O and $d_{O'}$ in the *compatible family* of distributions marginalize to the same disribution over the intersection of O with O'. If these constraints are not satisfied, then there is no way to make a consistent assignment of probabilities to the states of even a single variable. In this case in order to restore consistency one of the constraints must be eliminated or duplication of a variable may allow for the independent satisfaction of both constraints.

If, moreover, this first condition implies the existence of $d \in \mathcal{D}(\mathcal{E}(L))$ such that $d|O = d_O$ for all $O \in \mathcal{G}$ then the system is said to satisfy the global consistency condition. The space of all such globally consistent distributions for a given covering, $\mathcal{G}$, is referred to as $\mathbb{M}(\mathcal{G})$ where

$$\mathbb{M}(\mathcal{G}) = \{d_O \in \mathcal{D}(\mathcal{E}(\mathcal{G})) \mid (\exists d)\ d|O = d_O\}. \tag{S15}$$

In general, the system of equations $d|O = d_O$ for all $O \in \mathcal{G}$ is underdetermined and so local consistency does not imply global consistency. Local and global consistency are formalized as described in Sec. S3 in terms of sheaf theory as applied to the presheaf functors $\mathcal{E}$ and $\mathcal{D} \circ \mathcal{E}$. $\mathcal{E}$ alone turns out to be a sheaf because it satisfies the analogous conditions for all possible coverings $\mathcal{G}$ of L: for $\{e_O \in \mathcal{E}(O)|O \in \mathcal{G}\}$ such that $e_{O_1}|O_1 \cap O_2 = e_{O_2}|O_1 \cap O_2$ there exists a unique $e \in \mathcal{E}(\cup_{O\in\mathcal{G}} O)$ such that $e_O = e|O$ for all $O \in \mathcal{G}$. By analogy to Eq. S2 this is expressed by applying the same conditions to the equalizer diagram

$$\mathcal{E}(L) \overset{e}{\dashrightarrow} \coprod_{i\in I} \mathcal{E}(O_i) \underset{e_{O_j}}{\overset{e_{O_i}}{\rightrightarrows}} \coprod_{i,j\in I\times I} \mathcal{E}(O_{ij})\ . \tag{S16}$$

For $\mathcal{D} \circ \mathcal{E}$ the sheaf condition is not automatically satisfied and it only defines a presheaf. We examine the situation more closely to explicitly determine the necessary conditions for global consistency.

For a cover of the space of variables, $\mathcal{G}$, we can construct a linear operator, $\mathbf{G}$, representing the relationship, $R = \coprod_{O\in\mathcal{G}} \mathcal{E}(O \subset L) \subseteq \mathcal{E}(L) \times \mathcal{E}(\mathcal{G})$, between network-network state maps having as domain particular network modules given by the $O \in \mathcal{G}$ and those global network-network state maps defined on L. We would like to construct the matrix representation of $\mathbf{G}$. In the first factor, $\mathcal{E}(L) = P^L = \{e^L_{\vec{j}}|\vec{j} \in P^{|L|}\}$. For the second factor, $\mathcal{E}(\mathcal{G}) = \coprod_{O\in\mathcal{G}} \mathcal{E}(O) = \{e^O_{\vec{i}}|O \in \mathcal{G}, \vec{i} \in P^{|O|}\}$. So we have two sets of maps, one defined on P^L and the other defined on $\mathcal{E}(O) = P^O$ for each $O \in \mathcal{G}$. This yields the method of specifying the intended relationship that defines $\mathbf{G}$ for all $e^O_{\vec{i}} \in \mathcal{E}(\mathcal{G})$ and $e^L_{\vec{j}} \in \mathcal{E}(L)$ given in Eq. S17. This matrix can be viewed as an operator acting via matrix multiplication on distributions

$$\begin{aligned} \mathbf{G}\colon \mathcal{D}(\mathcal{E}(L)) &\rightarrow \mathcal{D}(\mathcal{E}(\mathcal{G})), \\ d &\mapsto \coprod_{O\in\mathcal{G}} d|O, \end{aligned}$$

and thereby taking a global distribution, $\mathcal{D}(\mathcal{E}(L))$, defined on network-network state maps whose domain is the full set of variables L into the local distributions, $\mathcal{D}(\mathcal{E}(\mathcal{G}))$, that are defined relative to network modules contained in a covering of the space of variables $\mathcal{G}$. $\mathbf{G}$ can be specified for all $e^O_{\vec{i}} \in \mathcal{E}(\mathcal{G})$ and $e^L_{\vec{j}} \in \mathcal{E}(L)$:

$$\mathbf{G}(e^O_{\vec{i}}, e^L_{\vec{j}}) = \begin{cases} 1, & e^L_{\vec{j}}|O = e^O_{\vec{i}}, \\ 0, & \text{otherwise.} \end{cases} \tag{S17}$$

For example, given the covering $\mathcal{G} = \{\{l_1\}, \{l_2\}\}$ of a set of two variables $L = \{l_1, l_2\}$ the associated matrix $\mathbf{G}$ is shown in Fig. S1B. $\mathbf{G}$ provides a way of determining the distributions on network-network

state maps for a given context (i.e. $\coprod_{O\in\mathcal{G}} d|O$) that can be derived from distributions (i.e. $\mathcal{D}(\mathcal{E}(L))$) defined on the global network-network state maps (i.e. $\mathcal{E}(L)$ as opposed to $\mathcal{E}(O)$).

Having expressed the relationship between global and local network-network state maps in terms of $\mathbf{G}$ we now make use of sheaf theory in order to extract the global consistency conditions. Given Eq. S16 and the associated conditions making $\mathcal{E}$ a sheaf, $\mathbb{R}^{\mathcal{E}}$ given by

$$\mathbb{R}^{\mathcal{E}(L)} \xrightarrow{\mathbf{G}} \bigoplus_{i\in I}\mathbb{R}^{\mathcal{E}(O_i)} \underset{\mathbf{H_2}}{\overset{\mathbf{H_1}}{\rightrightarrows}} \bigoplus_{i,j\in I\times I}\mathbb{R}^{\mathcal{E}(O_{ij})}\ , \tag{S18}$$

is a half-sheaf, in the sense that it satisfies the first two conditions but not the third uniqueness condition given in Sec. S3. It follows from this fact that $ker(\mathbf{H}_1 - \mathbf{H}_2) = im(\mathbf{G})$. Moreover, although $\mathcal{D}\circ\mathcal{E}$ is a mere presheaf, it can be embedded into $\mathbb{R}^{\mathcal{E}}$ using the map defined in Eq. S12 thereby allowing for the expression of consistency conditions on $\mathcal{D}\circ\mathcal{E}$ in terms of linear equations constituting constraints on the relevant probabilities. The following diagram demonstrates the relationships between the spaces of probability distributions and the linear spaces in which they are embedded:

$$\begin{array}{ccc} \mathbb{R}^{\mathcal{E}(L)} & \xrightarrow{\mathbf{G}} & \mathbb{R}^{\mathcal{E}(\mathcal{G})} \\ {\scriptstyle emb_{\mathcal{E}(L)}}\big\uparrow & & \big\uparrow{\scriptstyle emb_{\mathcal{E}(\mathcal{G})}} \\ \mathcal{D}(\mathcal{E}(L)) & \xrightarrow{\mathbf{G}} & \mathcal{D}(\mathcal{E}(\mathcal{G})) \end{array} \tag{S19}$$

The locally, $\mathbb{L}(\mathcal{G})$, and globally, $\mathbb{M}(\mathcal{G})$, consistent polytopes correspond to the spaces of probability distributions satisfying the local and global consistency conditions described above. In terms of the diagrams expressing the half-sheaf condition, Eq. S18, and embedding map, Eq. S19,

$$\begin{aligned} &\mathbb{M}(\mathcal{G}) = \mathbf{G}(emb_{\mathcal{E}(L)}(\mathcal{D}(\mathcal{E}(L)))) \\ &\mathbb{L}(\mathcal{G}) = emb_{\mathcal{E}(\mathcal{G})}(\mathcal{D}(\mathcal{E}(\mathcal{G}))) \cap ker(\mathbf{H}_1(\mathbb{R}^{\mathcal{E}(\mathcal{G})}) - \mathbf{H}_2(\mathbb{R}^{\mathcal{E}(\mathcal{G})})) = emb_{\mathcal{E}(\mathcal{G})}(\mathcal{D}(\mathcal{E}(\mathcal{G}))) \cap \mathbf{G}(\mathbb{R}^{\mathcal{E}(L)}). \end{aligned} \tag{S20}$$

As in Eq. S5

$$emb_{\mathcal{E}(\mathcal{G})}(\mathcal{D}(\mathcal{E}(\mathcal{G}))) = \left\{ p^O_{\vec{i}} \;\middle|\; (\forall\, O\in\mathcal{G})\ (\forall\,\vec{i}\in P^{|O|})\ \ p^O_{\vec{i}} \geq 0,\ (\forall\, O\in\mathcal{G}) \sum_{\vec{i}\in P^{|O|}} p^O_{\vec{i}} = 1 \right\}, \tag{S21}$$

$$emb_{\mathcal{E}(L)}(\mathcal{D}(\mathcal{E}(L))) = \left\{ p^L_{\vec{i}} \;\middle|\; (\forall\,\vec{i}\in P^{|L|})\ p^L_{\vec{i}} \geq 0,\ \sum_{\vec{i}\in P^{|L|}} p^L_{\vec{i}} = 1 \right\}. \tag{S22}$$

In general the globally consistent polytope is a proper subspace of the locally consistent one because $\mathbf{G}$ is not invertible (the maximum entropy principle is commonly used to make an arbitrary choice in the face of this underdetermination). To determine explicit conditions on the probabilities we express $\mathbb{L}(\mathcal{G})$ and $\mathbb{M}(\mathcal{G})$ in terms of the fundamental subspaces associated to the linear map $\mathbf{G}$. In order for a vector v to lie in $\mathbf{G}(\mathbb{R}^{\mathcal{E}(L)})$, we must have $v = \mathbf{G}x$ for some $x \in \mathbb{R}^{\mathcal{E}(L)}$. The cokernel of $\mathbf{G}$ gives the obstructions to this system $v = \mathbf{G}x$ having a solution. In order to eliminate these obstructions, constraints must be imposed on $\mathbb{R}^{\mathcal{E}(\mathcal{G})}$ and these constraints are given precisely via annihilating the cokernel, i.e. $\mathbf{G}(\mathbb{R}^{\mathcal{E}(L)}) = \{v \mid (\forall u \in coker\mathbf{G})\ u\cdot v = 0\}$. We then take the appropriate intersection to determine $\mathbb{L}(\mathcal{G})$ by requiring $v \in \mathcal{D}(\mathcal{E}(\mathcal{G}))$

$$\mathbb{L}(\mathcal{G}) = \{v \in \mathcal{D}(\mathcal{E}(\mathcal{G})) \mid (\forall u \in coker\mathbf{G})\ u\cdot v = 0\}. \tag{S23}$$

Since $\mathbf{G}$ is not invertible the equation $v = \mathbf{G}x$ can only be solved up to an element of $ker\mathbf{G}$. $v = \mathbf{G}x$ can thus be solved on a subspace T of $\mathbb{R}^{\mathcal{E}(L)}$ such that $T \oplus ker\mathbf{G} = \mathbb{R}^{\mathcal{E}(L)}$ to yield

$$\mathbb{M}(\mathcal{G}) = \{v \mid v = \mathbf{G}x,\ (\exists x\in T)\ (\exists y \in \mathcal{D}(\mathcal{E}(L)))\ x - y \in ker\mathbf{G}\}. \tag{S24}$$

If the embedding into linear spaces is to be considered explicitly, then Eq. S21 and Eq. S22 can be substituted for $\mathcal{D}(\mathcal{E}(\mathcal{G}))$ and $\mathcal{D}(\mathcal{E}(L))$ in Eq. S23 and Eq. S24. In order to obtain inequalities that define $\mathbb{M}(\mathcal{G})$, Fourier-Motzkin elimination can be used to eliminate x and y. Alternatively one can use the fact, [30] proposition 8.3, that $\mathbb{M}(\mathcal{G})$ is given by removing the non-integer vertices from a vertex representation of $\mathbb{L}(\mathcal{G})$ and the ability to interconvert between vertex and inequality representations to compute the same inequalities as described in Supplementary Material Sec. S6.

S5.1 Example of apparent satisfaction of unsatisfiable constraints

The inequalities defining $\mathbb{M}(\mathcal{G})$ were derived under the assumption that the two-element probabilities were obtained by mariginalizing a three-element distribution. If some other procedure, such as conditionalization, is used to obtain them instead, these inequalities need not apply. For example, suppose now that $L = \{l_1, l_2, l_3\}$, $P = \{0, 1, 2\}$, $\mathcal{G} = \{\{l_1, l_2\}, \{l_2, l_3\}, \{l_3, l_1\}\}$ where we have simply added an element to P relative to the example described above. In the previous example the marginal maps were given by $\mathcal{D}(\mathcal{E}(O \subset L))$ with one for each $O \in \mathcal{G}$. If we combine these marginal maps with conditioning on one out of the three variables being in state two and each of the other two being in states zero or one, then we have instead $\mathcal{D}(\pi_1), \mathcal{D}(\pi_2), \mathcal{D}(\pi_3)$ where $\pi_1 = \mathcal{E}(\{l_1, l_2\} \subset L)|\{e^{123}_{ij2} \mid i, j \in \{0, 1\}\}$, $\pi_2 = \mathcal{E}(\{l_2, l_3\} \subset L)|\{e^{123}_{2ij} \mid i, j \in \{0, 1\}\}$, $\pi_3 = \mathcal{E}(\{l_3, l_1\} \subset L)|\{e^{123}_{i2j} \mid i, j \in \{0, 1\}\}$. In this case, if we have the following assignment of probabilities for a distribution d

$$\begin{aligned}
p^{123}_{002} &= 1/30 & p^{123}_{020} &= 2/15 & p^{123}_{200} &= 2/15 \\
p^{123}_{012} &= 2/15 & p^{123}_{021} &= 1/30 & p^{123}_{201} &= 1/30 \\
p^{123}_{102} &= 2/15 & p^{123}_{120} &= 1/30 & p^{123}_{210} &= 1/30 \\
p^{123}_{112} &= 1/30 & p^{123}_{121} &= 2/15 & p^{123}_{211} &= 2/15
\end{aligned} \tag{S25}$$

with all other probabilities being zero, then $\mathcal{D}(\pi_1)(d), \mathcal{D}(\pi_2)(d), \mathcal{D}(\pi_3)(d)$ are equivalent to the probability tables in Fig. 3A, which as shown in Sec. 6, could not be achieved by marginalization alone. For example, given that $dom(\pi_1) = \{e^{123}_{002}, e^{123}_{012}, e^{123}_{102}, e^{123}_{112}\}$ then $d(dom(\pi_1)) = \frac{1}{30} + \frac{2}{30} + \frac{2}{30} + \frac{1}{30} = \frac{1}{3}$. Substituting this factor and the fact that ${\pi_1}^{-1}(e^{12}_{ij}) = e^{123}_{ij2}$ into Eq. S7

$$p^{12}_{ij} = \mathcal{D}(\pi_1)(d)(e^{12}_{ij}) = \frac{d({\pi_1}^{-1}(e^{12}_{ij}))}{d(dom(\pi_1))} = 3p^{123}_{ij2},$$

then renormalizes probabilities resulting in $p^{12}_{00} = 0.1, p^{12}_{01} = 0.4, p^{12}_{10} = 0.4, p^{12}_{11} = 0.1$ along with the analogs for p^{23}_{ij} and p^{13}_{ij}, which are precisely equivalent to what appears in Fig. 3A as suggested above.

If constraints consistent with those of Fig. 3A are placed on the given network, either the network must add another variable in order to satisfy them directly or the network context imposing those constraints must coarse-grain the network in a suitable way. In what follows, we argue that the former is much more plausible than the latter. This ultimately suggests conditions in which cycle breakage may be selected for to relieve inconsistent constraints that can arise when cycles are present.

S6 Example volume ratio computation for the four-cycle network architecture

For the purposes of this example, we take the full set of variables to be $L = \{l_1, l_2, l_3, l_4\}$. Consider the case in which each of the network modules under consideration has two variables and we specify the covering of the space of variables given by $\mathcal{G} = \{\{l_1, l_2\}, \{l_1, l_4\}, \{l_3, l_2\}, \{l_3, l_4\}\}$. We will compute $\mathbb{L}(\mathcal{G})$

using the same method which was used for the example of three variables. By analogy with Eq. 17, the local consistency conditions now are as follows:

$$\begin{aligned}
p^1_0 &= p^{12}_{00} + p^{12}_{01} = p^{14}_{00} + p^{14}_{01}, \quad p^1_1 = p^{12}_{10} + p^{12}_{11} = p^{14}_{10} + p^{14}_{11},\\
p^3_0 &= p^{32}_{00} + p^{32}_{01} = p^{34}_{00} + p^{34}_{01}, \quad p^3_1 = p^{32}_{10} + p^{32}_{11} = p^{34}_{11} + p^{34}_{11},\\
p^2_0 &= p^{12}_{00} + p^{12}_{10} = p^{32}_{00} + p^{32}_{10}, \quad p^2_1 = p^{12}_{01} + p^{12}_{11} = p^{32}_{01} + p^{32}_{11},\\
p^4_0 &= p^{14}_{00} + p^{14}_{10} = p^{34}_{00} + p^{34}_{10}, \quad p^4_1 = p^{14}_{01} + p^{14}_{11} = p^{34}_{01} + p^{34}_{11}.
\end{aligned} \tag{S26}$$

Likewise, the equations determined by the conditions $v = \mathbf{G}(x)$ which are analogous to the matrix $\mathbf{G}$ in Fig. S1B are now

$$\begin{aligned}
p^{12}_{00} &= p^{1234}_{0000} + p^{1234}_{0010} + p^{1234}_{0001} + p^{1234}_{0011} \quad & p^{12}_{10} &= p^{1234}_{1000} + p^{1234}_{1010} + p^{1234}_{1001} + p^{1234}_{1011}\\
p^{12}_{01} &= p^{1234}_{0100} + p^{1234}_{0110} + p^{1234}_{0101} + p^{1234}_{0111} \quad & p^{12}_{11} &= p^{1234}_{1100} + p^{1234}_{1110} + p^{1234}_{1101} + p^{1234}_{1111}\\
p^{14}_{00} &= p^{1234}_{0000} + p^{1234}_{0001} + p^{1234}_{1000} + p^{1234}_{1001} \quad & p^{14}_{10} &= p^{1234}_{0010} + p^{1234}_{0011} + p^{1234}_{1010} + p^{1234}_{1011}\\
p^{14}_{01} &= p^{1234}_{0100} + p^{1234}_{0101} + p^{1234}_{1100} + p^{1234}_{1101} \quad & p^{14}_{11} &= p^{1234}_{0110} + p^{1234}_{0111} + p^{1234}_{1110} + p^{1234}_{1111}\\
p^{32}_{00} &= p^{1234}_{0000} + p^{1234}_{0010} + p^{1234}_{0100} + p^{1234}_{0110} \quad & p^{32}_{10} &= p^{1234}_{1000} + p^{1234}_{1010} + p^{1234}_{1100} + p^{1234}_{1110}\\
p^{32}_{01} &= p^{1234}_{0001} + p^{1234}_{0011} + p^{1234}_{0101} + p^{1234}_{0111} \quad & p^{32}_{11} &= p^{1234}_{1001} + p^{1234}_{1011} + p^{1234}_{1101} + p^{1234}_{1111}\\
p^{34}_{00} &= p^{1234}_{0000} + p^{1234}_{1000} + p^{1234}_{0100} + p^{1234}_{1100} \quad & p^{34}_{10} &= p^{1234}_{0010} + p^{1234}_{1010} + p^{1234}_{0110} + p^{1234}_{1110}\\
p^{34}_{01} &= p^{1234}_{0001} + p^{1234}_{1001} + p^{1234}_{0101} + p^{1234}_{1101} \quad & p^{34}_{11} &= p^{1234}_{0011} + p^{1234}_{1011} + p^{1234}_{0111} + p^{1234}_{1111},
\end{aligned} \tag{S27}$$

which are displayed in matrix form in Table S1.

Rather than proceeding to compute $\mathbb{L}(\mathcal{G})$ using elimination of inequalities as before, we will instead make use of the fact that the extremal points of $\mathbb{L}(\mathcal{G})$ happen to be the extremal points of $\mathbb{L}(\mathcal{G})$ with integer coordinates. This is the approach which was used to compute the volume ratios shown in Fig. 5. More specifically, those computations were done using a computer program based on the following algorithm which is available via a virtual machine that can be reconstructed using the instructions available on github:

1. Compute (a basis for) the cokernel of $\mathbf{G}$. The cokernel gives the obstructions to the system $\mathbf{GX} = \mathbf{V}$ having a solution. In order to eliminate these obstructions constraints must be imposed on $\mathbb{R}^{\mathcal{E}(\mathcal{G})}$ and these constraints are given precisely via annihilating the cokernel.

2. Use the constraints on $\mathbb{R}^{\mathcal{E}(\mathcal{G})}$ from step 1 necessary for the system $\mathbf{GX} = \mathbf{V}$ to have a solution to eliminate variables from the system of inequalities $\mathbf{V} \geq 0$ giving a half-space representation or H-representation of the polytope $\mathbb{L}(\mathcal{G})$. This can be used to compute $\mathrm{Vol}(\mathbb{L}(\mathcal{G}))$.

3. Compute the vertices of $\mathbb{L}(\mathcal{G})$ from the H-representation determined in step 2 giving a vertex representation or V-representation of $\mathbb{L}(\mathcal{G})$.

4. Filter the non-integer rational vertices from the collection computed in step 3 to produce a corresponding V-representation of $\mathbb{M}(\mathcal{G})$ [30] proposition 8.3.

5. Compute $\mathrm{Vol}(\mathbb{M}(\mathcal{G}))$ from the V-represention of $\mathbb{M}(\mathcal{G})$.

For standard computations on polytopes, we make use of the standard algorithms incorporated by the polymake project [52]. In some cases, the volume computation is too costly to perform exactly. In those cases we use the approximation given in [53]. We now return to our example of four variables $\mathcal{G} = \{\{l_1, l_2\}, \{l_1, l_4\}, \{l_3, l_2\}, \{l_3, l_4\}\}$ and $P = \{0, 1\}$ and use it to walk through key components of the algorithm.

The equalities derived by computing the cokernel of the matrix $\mathbf{G}$ given in Table S1 and adjoining rows that enforce the normalization of the marginal distributions are represented as a matrix in Eq. S28.

$$\begin{bmatrix}
-1 & -1 & 0 & 0 & 1 & 1 & 0 & 0 & 0 & 0 & 0 & 0 & 0 & 0 & 0 & 0 & 0 \\
0 & 0 & -1 & -1 & 0 & 0 & 1 & 1 & 0 & 0 & 0 & 0 & 0 & 0 & 0 & 0 & 0 \\
-1 & 0 & -1 & 0 & 0 & 0 & 0 & 0 & 1 & 1 & 0 & 0 & 0 & 0 & 0 & 0 & 0 \\
0 & -1 & 0 & -1 & 0 & 0 & 0 & 0 & 0 & 0 & 1 & 1 & 0 & 0 & 0 & 0 & 0 \\
0 & 0 & 0 & 0 & 0 & 0 & 0 & 0 & -1 & 0 & -1 & 0 & 1 & 1 & 0 & 0 & 0 \\
0 & 0 & 0 & 0 & -1 & 0 & -1 & 0 & 0 & 0 & 0 & 0 & 1 & 0 & 1 & 0 & 0 \\
-1 & -1 & -1 & -1 & 1 & 0 & 1 & 0 & 1 & 0 & 1 & 0 & -1 & 0 & 0 & 1 & 0 \\
1 & 1 & 1 & 1 & 0 & 0 & 0 & 0 & 0 & 0 & 0 & 0 & 0 & 0 & 0 & 0 & 1 \\
0 & 0 & 0 & 0 & 1 & 1 & 1 & 1 & 0 & 0 & 0 & 0 & 0 & 0 & 0 & 0 & 1 \\
0 & 0 & 0 & 0 & 0 & 0 & 0 & 0 & 1 & 1 & 1 & 1 & 0 & 0 & 0 & 0 & 1 \\
0 & 0 & 0 & 0 & 0 & 0 & 0 & 0 & 0 & 0 & 0 & 0 & 1 & 1 & 1 & 1 & 1
\end{bmatrix} \tag{S28}$$

The final column represents the right-hand side of each equality. It turns out all but one of the normalization conditions is linearly dependent with respect to the other equalities and so we can reduce this set of $7 + 4 = 11$ constraints to the 8 represented again in matrix form in Eq. S29.

$$\begin{bmatrix}
1 & 0 & 0 & -1 & 0 & 0 & 1 & 1 & 0 & 0 & 1 & 1 & 0 & 0 & 0 & 0 & 1 \\
0 & 1 & 0 & 1 & 0 & 0 & 0 & 0 & 0 & 0 & -1 & -1 & 0 & 0 & 0 & 0 & 0 \\
0 & 0 & 1 & 1 & 0 & 0 & -1 & -1 & 0 & 0 & 0 & 0 & 0 & 0 & 0 & 0 & 0 \\
0 & 0 & 0 & 0 & 1 & 0 & 1 & 0 & 0 & 0 & 0 & 0 & 0 & 1 & 0 & 1 & 1 \\
0 & 0 & 0 & 0 & 0 & 1 & 0 & 1 & 0 & 0 & 0 & 0 & 0 & -1 & 0 & -1 & 0 \\
0 & 0 & 0 & 0 & 0 & 0 & 0 & 0 & 1 & 0 & 1 & 0 & 0 & 0 & 1 & 1 & 1 \\
0 & 0 & 0 & 0 & 0 & 0 & 0 & 0 & 0 & 1 & 0 & 1 & 0 & 0 & -1 & -1 & 0 \\
0 & 0 & 0 & 0 & 0 & 0 & 0 & 0 & 0 & 0 & 0 & 0 & 1 & 1 & 1 & 1 & 1
\end{bmatrix} \tag{S29}$$

These equalities can now be substituted into the positivity inequalities necessary to define any space of probability distributions. This yields a set of inequalities Eq. S30 that specify an H-representation of the polytope $\mathbb{L}(\mathcal{G})$. This is the modular polytope, which is a subspace of $\Delta_3^{\oplus 4}$ associated to distributions consistent with the linear transformation $\mathbf{G}$

$$\begin{bmatrix}
1 & 1 & -1 & -1 & -1 & -1 & 0 & 0 & 0 \\
0 & -1 & 0 & 0 & 1 & 1 & 0 & 0 & 0 \\
0 & -1 & 1 & 1 & 0 & 0 & 0 & 0 & 0 \\
1 & 0 & -1 & 0 & 0 & 0 & -1 & 0 & -1 \\
0 & 0 & 0 & -1 & 0 & 0 & 1 & 0 & 1 \\
1 & 0 & 0 & 0 & -1 & 0 & 0 & -1 & -1 \\
0 & 0 & 0 & 0 & 0 & -1 & 0 & 1 & 1 \\
1 & 0 & 0 & 0 & 0 & 0 & -1 & -1 & -1 \\
0 & 1 & 0 & 0 & 0 & 0 & 0 & 0 & 0 \\
0 & 0 & 1 & 0 & 0 & 0 & 0 & 0 & 0 \\
0 & 0 & 0 & 1 & 0 & 0 & 0 & 0 & 0 \\
0 & 0 & 0 & 0 & 1 & 0 & 0 & 0 & 0 \\
0 & 0 & 0 & 0 & 0 & 1 & 0 & 0 & 0 \\
0 & 0 & 0 & 0 & 0 & 0 & 1 & 0 & 0 \\
0 & 0 & 0 & 0 & 0 & 0 & 0 & 1 & 0 \\
0 & 0 & 0 & 0 & 0 & 0 & 0 & 0 & 1
\end{bmatrix} \tag{S30}$$

A row $(a_0, a_1, ..., a_d)$ corresponds to the inequality $a_0 + a_1 x_1 + ... + a_d x_d >= 0$. The embedded identity matrix has, in this particular case eight, rows that specify the positivity of the variables corresponding

to each of the, in this particular case eight, dimensions. Transforming this inequality or H-representation to a vertex or V-representation of the modular polytope produces Eq. S31.

$$\begin{bmatrix}
1 & 0 & 0 & 0 & 0 & 0 & 0 & 0 & 1 \\
1 & 0 & 0 & 0 & 0 & 0 & 0 & 1 & 0 \\
1 & 0 & 0 & 0 & 0 & 0 & 1 & 0 & 0 \\
1 & 1/2 & 1/2 & 0 & 1/2 & 0 & 1/2 & 1/2 & 0 \\
1 & 1/2 & 0 & 1/2 & 0 & 1/2 & 1/2 & 1/2 & 0 \\
1 & 0 & 0 & 0 & 0 & 0 & 0 & 0 & 0 \\
1 & 1/2 & 1/2 & 0 & 0 & 1/2 & 0 & 0 & 1/2 \\
1 & 1/2 & 0 & 1/2 & 1/2 & 0 & 0 & 0 & 1/2 \\
1 & 0 & 0 & 1/2 & 0 & 1/2 & 0 & 0 & 1/2 \\
1 & 0 & 1/2 & 0 & 1/2 & 0 & 0 & 0 & 1/2 \\
1 & 0 & 1/2 & 0 & 0 & 1/2 & 1/2 & 1/2 & 0 \\
1 & 0 & 0 & 1/2 & 1/2 & 0 & 1/2 & 1/2 & 0 \\
1 & 1 & 1 & 0 & 1 & 0 & 0 & 0 & 0 \\
1 & 0 & 0 & 0 & 1 & 0 & 0 & 0 & 0 \\
1 & 0 & 1 & 0 & 0 & 0 & 0 & 0 & 0 \\
1 & 0 & 0 & 1 & 0 & 0 & 1 & 0 & 0 \\
1 & 0 & 0 & 0 & 0 & 1 & 0 & 1 & 0 \\
1 & 0 & 0 & 0 & 1 & 0 & 1 & 0 & 0 \\
1 & 0 & 1 & 0 & 0 & 0 & 0 & 1 & 0 \\
1 & 1 & 0 & 1 & 0 & 1 & 0 & 0 & 1 \\
1 & 1 & 0 & 1 & 1 & 0 & 1 & 0 & 0 \\
1 & 1 & 1 & 0 & 0 & 1 & 0 & 1 & 0 \\
1 & 0 & 0 & 1 & 0 & 0 & 0 & 0 & 1 \\
1 & 0 & 0 & 0 & 0 & 1 & 0 & 0 & 1
\end{bmatrix} \tag{S31}$$

This completes steps 1-3 of the algorithm outlined above. Step 4 is trivial; to obtain the V-represention of $\mathbb{M}(\mathcal{G})$, we strike out the rows in which 1/2 appears. Finally, we compute the volume of the polytope whose vertices are the rows of Eq. S31 to obtain $\mathrm{Vol}(\mathbb{L}(\mathcal{G})) = \frac{1}{120}$ and the volume of the polytope whose vertices are rows of integers to obtain $\mathrm{Vol}(\mathbb{M}(\mathcal{G})) = \frac{1}{180}$ yielding a ratio $\frac{\mathrm{Vol}(\mathbb{M}(\mathcal{G}))}{\mathrm{Vol}(\mathbb{L}(\mathcal{G}))} = \frac{2}{3}$.

A

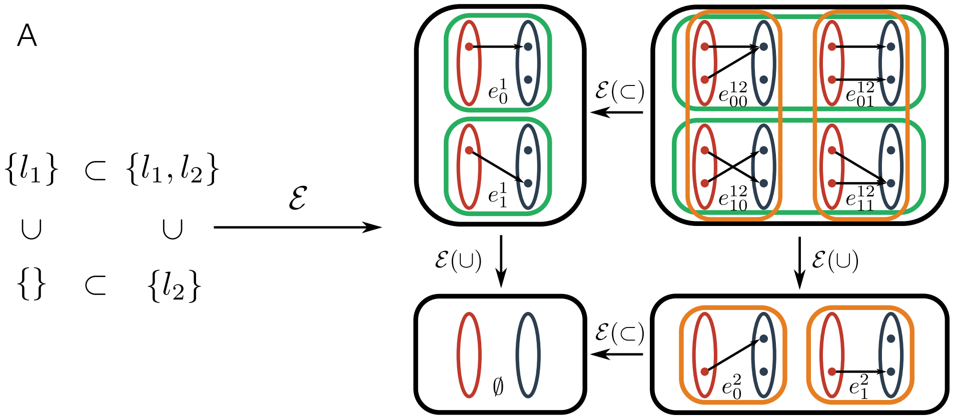

B

$$\mathcal{G} = \{\{l_1\}, \{l_2\}\} \Longrightarrow \mathbf{G} =$$

	e^{12}_{00}	e^{12}_{01}	e^{12}_{10}	e^{12}_{11}
e^1_0	1	1	0	0
e^1_1	0	0	1	1
e^2_0	1	0	1	0
e^2_1	0	1	0	1

Figure S1. Example of the functor mapping subsets of variables to measurable spaces. (A) On the left hand side are subsets of $L = \{l_1, l_2\}$ ordered by inclusion. On the right hand side are the spaces of network-network state maps also ordered by inclusion. The labels for the maps define them. For example, $e^{12}_{01}(l_1) = 0$ and $e^{12}_{01}(l_2) = 1$. (B) For the given covering, $\mathcal{G}$, the associated marginalization matrix acting on the probability vector $\{p^{12}_{00}, p^{12}_{01}, p^{12}_{10}, p^{12}_{11}\}$ to give $\{p^1_0, p^1_1, p^2_0, p^2_1\}$ is $\mathbf{G}$.

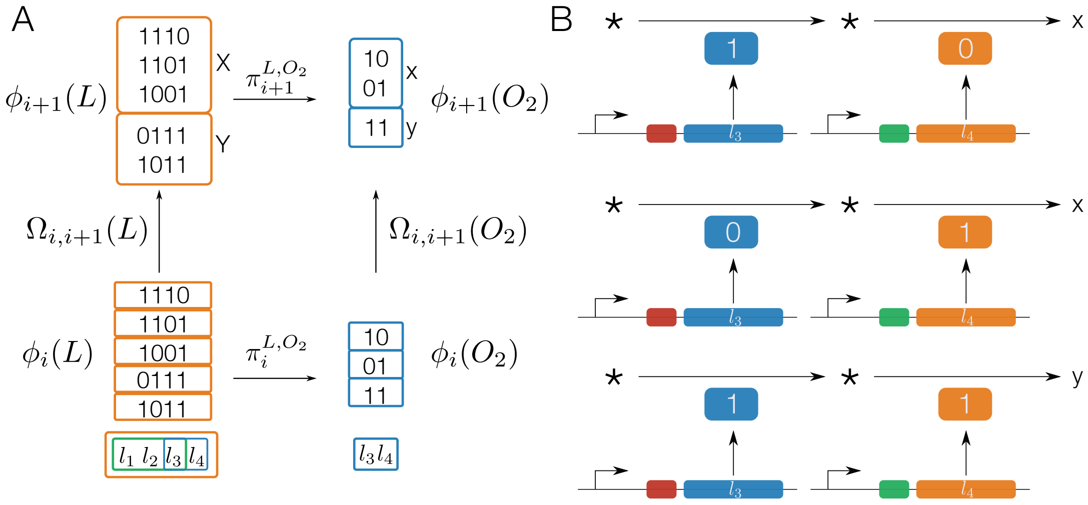

Figure S2. Example coarse-graining of phenotypes. (A) Consider the example where $L = \{l_1, l_2, l_3, l_4\}$, $\mathcal{G} = \{O_1, O_2\}$, $O_1 = \{l_1, l_2, l_3\}$ and $O_2 = \{l_3, l_4\}$. The top left panel shows two higher-level phenotypes X and Y. The bottom left corner shows the five different expression states of four genes in L from which these phenotypes are coarse-grained. The right side shows the respective projections onto genes $\{l_3, l_4\}$. The projection maps π_i^{L,O_2} and π_{i+1}^{L,O_2} are defined in Supplementary Material Sec. S4. (B) The different combinations of expression states of genes $\{l_3, l_4\}$ result in two different phenotypes. If both genes are expressed metabolite y is produced whereas if only one of the two genes is expressed metabolite x is produced. The red and green boxes represent arbitrary promoters.

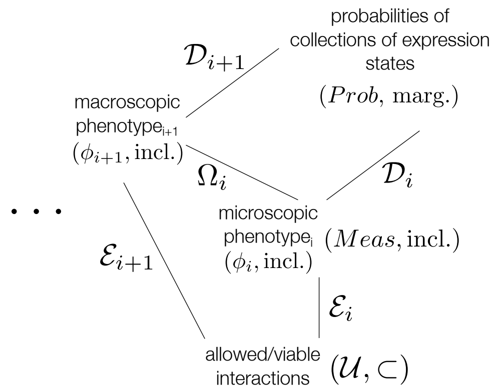

Figure S3. Mathematical relationships defining the hierarchy of network states via coarse-graining.

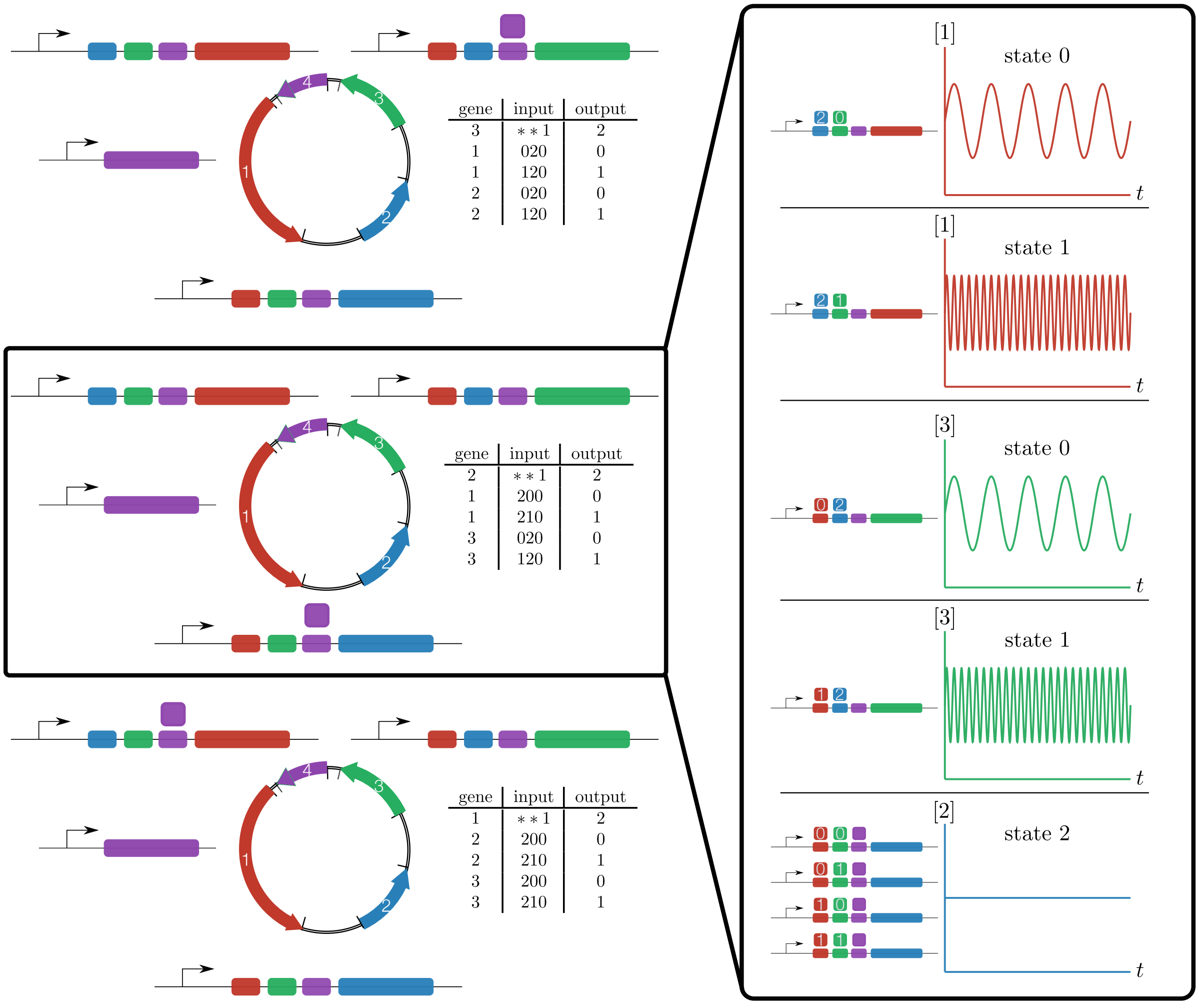

Figure S4. Schematic synthetic gene circuit capable of exhibiting apparent inconsistency. A synthetic gene circuit consisting of four genes possesses one gene (purple) whose product is assumed to be present at low copy number and binds randomly with equal affinity to operator sites existing within the operons of each of the other three genes (red, blue, and green). These latter three genes each possess operator sites for the other two, but do not possess autoregulatory operators. They also each exhibit three states represented by three dynamical modes that may involve intermediates not explicitly represented here [54, 55]. If the first gene is bound to the operator of another gene, the output is forced into a zero frequency infinite period, or DC, mode (state 2) regardless of the binding state of the other operators. If the first gene is unbound, then the expression state can be switched between low (state 0) and high (state 1) frequency modes depending upon the binding states of other genes as indicated. Note that operators for each of genes one to three are insensitive to the DC mode. Observing pairs of genes one to three and ignoring the state corresponding to the DC mode can lead to apparent inconsistency.

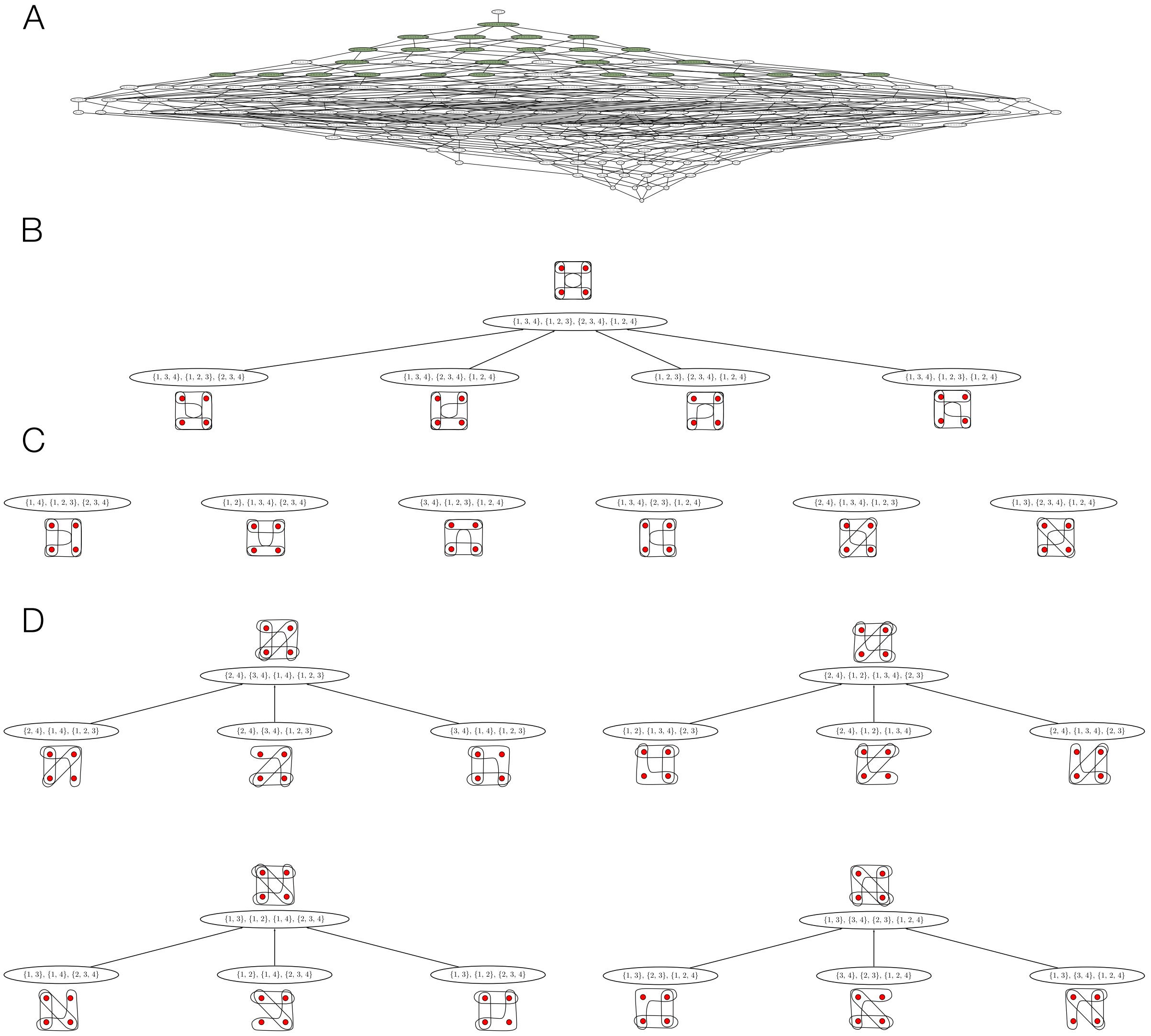

Figure S5. Hierarchical relationships among all possible classes of hypergraphs that are not graphs (i.e. not 2-uniform) but have cycles. (A) There is a Hasse diagram for the lattice of network architectures analogous to that of Fig. 4A but defined on four rather than only three variables. Within this lattice some of the graphs have cycles and some do not. (B) The highest levels of the Hasse diagram associated to the lattice of network architectures on four variables containing hypergraphs having cycles. (C) and (D) contain lower levels of network architectures containing cycles. Each of the four panels in (D) are on the same level. In total, each level represents an isomorphism class of hypergraphs. Therefore, there are five isomorphism classes of non-2-uniform hypergraphs representing network architectures on four variables that contain cycles leading to the relationship between spaces of probability distsributions on associated genotype-phentoype maps analogous to that of Fig. 4C.

	e^{1234}_{0000}	e^{1234}_{0010}	e^{1234}_{0001}	e^{1234}_{0011}	e^{1234}_{1000}	e^{1234}_{1010}	e^{1234}_{1001}	e^{1234}_{1011}	e^{1234}_{0100}	e^{1234}_{0110}	e^{1234}_{0101}	e^{1234}_{0111}	e^{1234}_{1100}	e^{1234}_{1110}	e^{1234}_{1101}	e^{1234}_{1111}
e^{12}_{00}	1	1	1	1	0	0	0	0	0	0	0	0	0	0	0	0
e^{12}_{10}	0	0	0	0	1	1	1	1	0	0	0	0	0	0	0	0
e^{12}_{01}	0	0	0	0	0	0	0	0	1	1	1	1	0	0	0	0
e^{12}_{11}	0	0	0	0	0	0	0	0	0	0	0	0	1	1	1	1
e^{32}_{00}	1	0	1	0	1	0	1	0	0	0	0	0	0	0	0	0
e^{32}_{10}	0	1	0	1	0	1	0	1	0	0	0	0	0	0	0	0
e^{32}_{01}	0	0	0	0	0	0	0	0	1	0	1	0	1	0	1	0
e^{32}_{11}	0	0	0	0	0	0	0	0	0	1	0	1	0	1	0	1
e^{14}_{00}	1	1	0	0	0	0	0	0	1	1	0	0	0	0	0	0
e^{14}_{10}	0	0	0	0	1	1	0	0	0	0	0	0	1	1	0	0
e^{14}_{01}	0	0	1	1	0	0	0	0	0	0	1	1	0	0	0	0
e^{14}_{11}	0	0	0	0	0	0	1	1	0	0	0	0	0	0	1	1
e^{34}_{00}	1	0	0	0	1	0	0	0	1	0	0	0	1	0	0	0
e^{34}_{10}	0	1	0	0	0	1	0	0	0	1	0	0	0	1	0	0
e^{34}_{01}	0	0	1	0	0	0	1	0	0	0	1	0	0	0	1	0
e^{34}_{11}	0	0	0	1	0	0	0	1	0	0	0	1	0	0	0	1

Table S1. Explicit construction of $\mathbf{G}_{n\times m}$ for the case $L = \{l_1, l_2, l_3, l_4\}$, $\mathcal{G} = \{\{l_1, l_2\}, \{l_1, l_4\}, \{l_3, l_2\}, \{l_3, l_4\}\}$, $P = \{0, 1\}$ and thus $\mathbf{G}_{(2\cdot 2)^2\times 2^{2\cdot 2}} = \mathbf{G}_{16\times 16}$.
